# Wolfram Syndrome protein, Miner1, regulates sulphydryl redox status, the unfolded protein response, and Ca^2+^ homeostasis

**DOI:** 10.1002/emmm.201201429

**Published:** 2013-05-24

**Authors:** Sandra E Wiley, Alexander Y Andreyev, Ajit S Divakaruni, Robert Karisch, Guy Perkins, Estelle A Wall, Peter van der Geer, Yi-Fan Chen, Ting-Fen Tsai, Melvin I Simon, Benjamin G Neel, Jack E Dixon, Anne N Murphy

**Affiliations:** 1 Department of Pharmacology, University of CaliforniaSan Diego, La Jolla, CA, USA; 2 Campbell Family Cancer Research Institute, Ontario Cancer Institute, Princess Hospital, University Health Network and Department of Medical Biophysics, University of TorontoToronto, ON, Canada; 3 National Center for Imaging Research, University of CaliforniaSan Diego, La Jolla, CA, USA; 4 Department of Chemistry and Biochemistry, San Diego State UniversitySan Diego, CA, USA; 5 Department of Life Sciences and Institute of Genome Sciences, National Yang-Ming UniversityTaipei, Taiwan; 6 Department of Chemistry and Biochemistry, University of CaliforniaSan Diego, La Jolla, CA, USA; 7 Department of Cellular and Molecular Medicine, University of CaliforniaSan Diego, La Jolla, CA, USA; 8 Howard Hughes Medical InstituteChevy Chase, MD, USA

**Keywords:** calcium, endoplasmic reticulum, mitochondria, oxidative stress, Wolfram Syndrome

## Abstract

Miner1 is a redox-active 2Fe2S cluster protein. Mutations in Miner1 result in Wolfram Syndrome, a metabolic disease associated with diabetes, blindness, deafness, and a shortened lifespan. Embryonic fibroblasts from Miner1^−/−^ mice displayed ER stress and showed hallmarks of the unfolded protein response. In addition, loss of Miner1 caused a depletion of ER Ca^2+^ stores, a dramatic increase in mitochondrial Ca^2+^ load, increased reactive oxygen and nitrogen species, an increase in the GSSG/GSH and NAD^+^/NADH ratios, and an increase in the ADP/ATP ratio consistent with enhanced ATP utilization. Furthermore, mitochondria in fibroblasts lacking Miner1 displayed ultrastructural alterations, such as increased cristae density and punctate morphology, and an increase in O_2_ consumption. Treatment with the sulphydryl anti-oxidant *N*-acetylcysteine reversed the abnormalities in the Miner1 deficient cells, suggesting that sulphydryl reducing agents should be explored as a treatment for this rare genetic disease.

## INTRODUCTION

Wolfram Syndrome (DIDMOAD) is an incurable disease characterized by a range of endocrine and neurological symptoms, including diabetes insipidus, diabetes mellitus, blindness due to optic atrophy and sensorineural deafness. Wolfram Syndrome is also associated with an increased incidence of psychiatric disorders and a significantly shortened life span, averaging only 30 years (Barrett et al, 1995; Strom et al, 1998). Two unrelated genes have been shown to be mutated in Wolfram Syndrome (Amr et al, 2007; Sam et al, 2001). These genes, originally designated as *WFS1* and *WFS2*, have since been demonstrated to encode the Wolframin and Miner1 proteins.

Mutations in Wolframin (*WFS1*), the first gene linked to Wolfram Syndrome, are responsible for the majority of Wolfram Syndrome cases. Recently, single nucleotide polymorphisms (SNPs) of the *WFS1* gene have also been implicated in the pathogenesis of type 2 diabetes (Wasson & Permutt, 2008). *WFS1* encodes a 100 kDa integral membrane protein of the ER that lacks known catalytic domains (Hofmann, 2003). Although there is some evidence that WFS1 is involved in Ca^2+^ homeostasis and influences the stability of the ER stress sensor ATF6 (Fonseca et al, 2010; Osman, 2003; Takei et al, 2006), the exact function of WFS1, its regulation, and the molecular mechanisms linking its function to Wolfram Syndrome and type 2 diabetes are far from resolved.

WFS2 encodes the Miner1 protein (aka: ERIS, CISD2). We previously identified a small family of proteins with high sequence similarity that includes mitoNEET, Miner1 and Miner2 (Wiley et al, 2007a). The current names for the genes encoding these proteins are *CISD* (CDGSH Iron Sulphur Domain) *1*, *2* and *3*, respectively. CDGSH domains are characterized by the presence of a Cys-Asp-Gly-Ser-His motif. MitoNEET and Miner1 each contain a single carboxy-terminal CDGSH domain, while Miner2 possesses tandem CDGSH domains. In addition to the CDGSH domain, there is a predicted hydrophobic sequence at the amino-terminus of Miner1. The CDGSH domain binds a redox active 2Fe2S cluster that is coordinated by an uncommon arrangement of 3 Cys and 1 His (Wiley et al, 2007b). The structures of the CDGSH domains of mitoNEET and Miner1 are virtually identical (Conlan et al, 2009; Paddock et al, 2007). The molecules exist as dimers with a unique fold. The His that ligates the 2Fe2S cluster is surface exposed and exquisitely sensitive to protonation if the pH is less than 7.0, resulting in release of the 2Fe2S cluster, a unique property among FeS cluster-binding proteins. This feature has led to the speculation that this family of proteins may be involved in FeS cluster assembly or their mobilization within the cell; alternatively, it may function in a redox capacity, as has been found for other 2Fe2S cluster proteins. Our initial data suggested that Miner1 with a C-terminal EGFP tag localized to the ER (Wiley et al, 2007a). However, a recent study concluded that Miner1 is predominantly a mitochondrial protein (Chen et al, 2009).

In an effort to locate a previously mapped longevity gene linked to human chromosome 4q (Puca et al, 2001), Tsai and colleagues generated mice in which the Miner1 (*Cisd2*) gene is deleted (Chen et al, 2009). These mice have a complex and dramatic phenotype and, indeed, appear to be a remarkable model of early aging, in addition to recapitulating many of the features of Wolfram Syndrome. Within 3 weeks after birth, pups lacking Miner1 show signs of optic and sciatic nerve atrophy. These mice develop normally for a short period and then proceed to display sarcopenia, thinning of the subcutaneous fat, hair loss, hair greying, osteopenia, lordokyphosis and significantly shortened lifespan. Although *Cisd2* KO mice are not overtly diabetic, their glucose tolerance is impaired.

Our understanding of the biological function of CDGSH domain proteins is still in its infancy. The phenotype of the *Cisd2* KO mice suggests that Miner1 is crucial for the maintenance of multiple organ systems throughout the body, including the pancreas, skin, musculoskeletal and nervous systems. Miner1 appears to be at the nexus of metabolism and lifespan control. Insights into the functions of Miner1 will not only provide knowledge regarding the etiology of Wolfram Syndrome, but should also shed light on an important new regulatory protein linking metabolic disease and aging.

Given the importance of ER/mitochondrial interactions to metabolic regulation, we have used mouse embryonic fibroblasts (MEFs) derived from Miner1 WT and KO mice to investigate the role of Miner1 in maintaining proper ER function and ER-mitochondrial communication. Miner1 KO cells displayed a dramatic reduction in ER Ca^2+^ and profound mitochondrial Ca^2+^ loading. Although mitochondrial respiratory capacity was increased in the KO cells, there was an increase in the ADP/ATP ratio and impaired cell proliferation. Miner1 deficient cells also displayed signs of oxidative stress and initiation of the unfolded protein response (UPR). Remarkably, treatment with the anti-oxidant *N*-acetylcysteine (NAC) reversed many of the molecular abnormalities caused by Miner1 deletion. Current treatments for Wolfram Syndrome focus on managing the diabetic symptoms; however, none of these reverse the degenerative course of the disease. Our data suggest that a potential therapeutic approach utilizing sulphydryl anti-oxidants, such as NAC, could more effectively target the underlying cause of the disease.

## RESULTS

### Miner1 is an integral membrane protein that localizes to the ER and MAMs facing the cytosol

The conflicting conclusions regarding the localization of Miner1 (ER *versus* mitochondria) may stem from the considerable physical contact between the mitochondria and the ER (Pizzo & Pozzan, 2007). Mitochondria-associated ER membranes (MAMs) consist of ER and mitochondrial proteins and represent regions of direct physical contact between the two organelles, typically rich in proteins involved in Ca^2+^ signalling and lipid biosynthesis (Osman et al, 2011; Zampese et al, 2011).

Because the ER, mitochondria and MAMs perform very distinct functions, defining the exact localization of Miner1 within the cell is an important and necessary first step towards understanding its role in Wolfram Syndrome.

**Figure 1:**
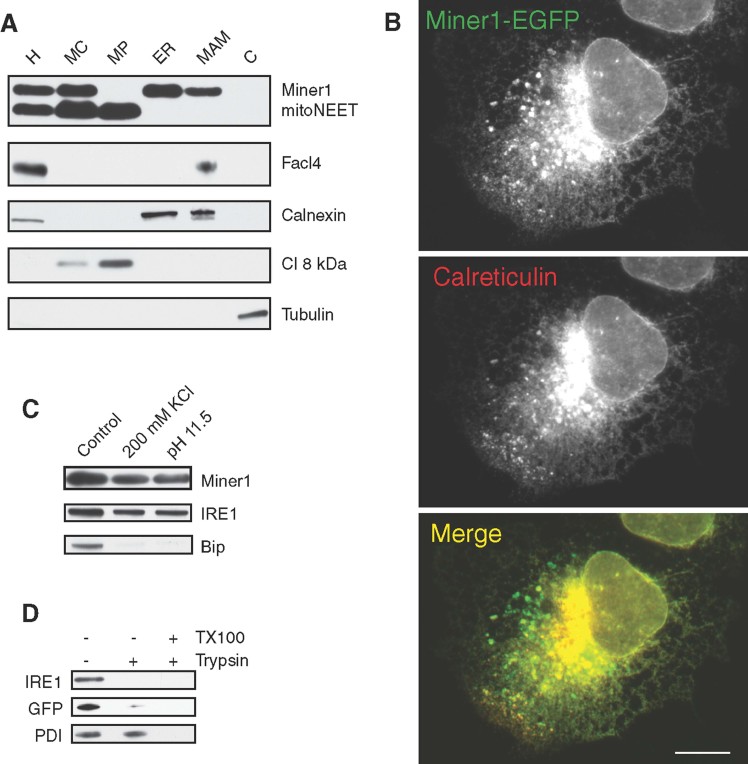
Miner1 is an ER and MAM integral membrane protein facing the cytosol Source data is available for this figure in the Supporting Information. Western blot analysis of rat liver subcellular fractions (30 µg protein per lane), immunoblotted with antibodies to marker proteins: mitochondrial (mitoNEET and Complex I 8 kDa protein), ER (calnexin), MAMs (FACL4), cytosolic/soluble proteins (tubulin), H = post-nuclear homogenate; MC = crude mitochondria; MP = pure mitochondria; ER = endoplasmic reticulum; MAM = mitochondrial associated ER membranes; C = cytosol.Immunofluorescent imaging of transiently transfected COS-7 cells expressing Miner1-EGFP (green), costained with anti-calreticulin (red). These are shown in black and white. In the merged pseudocolored image, the co-localized portion appears yellow. Scale bar is 10 µm.Western blot analysis of rat liver ER microsomes after treatment with either high salt (200 mM KCl) or alkaline (0.1 M Na_2_CO_3_, pH 11.5) to remove peripheral membrane proteins. IRE1 (an integral membrane protein) and Bip (a peripheral membrane associated protein) were included as controls.Western blot analysis of HEK293T cells transiently transfected with Miner1-EGFP. Microsomes were prepared and treated with trypsin ± 1% Triton X100 (TX100). The disappearance of the Miner1-EGFP fusion protein (46 kDa) was monitored using an antibody to GFP. IRE1 immunoreactivity was used as a control for an integral ER protein oriented towards the cytosol, and PDI as a control for a protein present within the ER lumen.

To this end, we isolated microsomal (ER) fractions, MAMs and mitochondria from rat livers and evaluated them by Western blotting with antibodies recognizing marker proteins to the various fractions: Miner1, ER (calnexin), MAM (FACL4), cytosol (tubulin) and mitochondria (Complex I 8 kDa protein). Our data revealed that Miner1 was most abundant in the ER-enriched fractions and was not detected in highly purified mitochondria (MP) (Fig 1A). It is noteworthy that there was a substantial amount of Miner1 present in the MAM fraction. To confirm the ER localization, we used fluorescence microscopy. C-terminally tagged Miner1-EGFP displayed a strong perinuclear localization that extended into a lacy reticulum present throughout the cell (Fig 1B). This pattern is typical of ER proteins such as calreticulin (Fig 1B). We have previously reported that Miner1 does not colocalize with the mitochondrial marker MitoTracker Red (Wiley et al, 2007a). A triple staining of cells with Miner1-EGFP, an ER marker and MitoTracker Red further demonstrates the positive co-localization of Miner1 with the ER marker and lack of mitochondrial localization (Supporting Information Figs S1, S2).

While we have clearly established that Miner1 localizes to the ER, it remains unknown how it is anchored there and whether the CDGSH domain is present in the ER lumen or the cytoplasm. To distinguish integral from peripheral membrane proteins, microsomes were subjected to high salt and alkaline washes. Miner1 remained associated with microsomal membranes under these conditions, as did the ER integral membrane protein IRE1 (Fig 1C). This is in contrast to Bip, a peripheral membrane-associated protein of the ER lumen. This establishes Miner1 as an integral membrane protein of the ER, which is consistent with the Octopus program predictions of a transmembrane (TM) domain between amino acids 37–57 in human Miner1 (Viklund & Elofsson, 2008). We performed trypsinolysis on freshly prepared microsomes containing Miner1-EGFP with a trypsin cleavage site in the linker region between the two proteins. The integrity of the full-length Miner1-EGFP protein was monitored by anti-GFP Western blotting. In the absence of detergents, Miner1-EGFP was susceptible to trypsinolysis, as was the IRE1 protein, an integral ER membrane kinase with the bulk of the protein facing the cytosol, in contrast to the luminal protein disulphide isomerase (PDI) (Fig 1D). Taken together, our data define Miner1 as an integral ER membrane protein with the CDGSH domain oriented towards the cytosol.

### Miner1 KO MEFs show signs of ER stress and UPR

Wolfram Syndrome has been linked to mutations in both *WFS1* and *CISD2* (Miner1). It has been reported that deletion of *Wfs1* results in ER stress and the initiation of the UPR (Fonseca et al, 2010; Yamada, 2006). Thus, we hypothesized that deletion of the Miner1 gene would also lead to an induction of the UPR and that this is a common thread in the etiology of Wolfram Syndrome.

**Figure 2:**
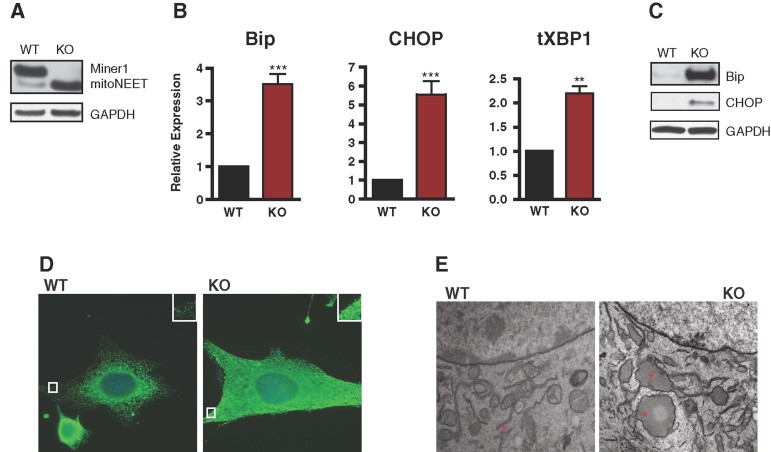
Deletion of Miner1 leads to ER stress Source data is available for this figure in the Supporting Information. Western blot analysis of whole cell lysates from wildtype (WT) and Miner1 knockout (KO) MEFs (20 µg protein).qRT-PCR of the ER stress markers Bip (*t*-test, *n* = 6, *p* < 0.0001), CHOP (*t*-test, *n* = 6, *p* < 0.0001), and total XBP1 (*t*-test, *n* = 3, *p* = 0.0013), mean ± SE.Western blot analysis of whole cell lysates from Miner1 WT and KO MEFs (15 µg protein).Immunofluorescent staining of WT and KO MEFs using an anti-calreticulin (ER, green) antibody and DAPI (nucleus, blue). Insets are 300% enlargements of small boxes (positioned over plasma membrane and subplasmalemmal area). Scale bars are 10 µm.Electron micrographs of WT and KO MEFs revealing swollen ER in KO cells. Normal *versus* distended portions of the ER are marked with a red asterisk in the images of WT and KO, respectively.

Taking advantage of the recent development of a Miner1 (*Cisd2*) KO mouse, we immortalized early passage primary MEF cells from mutant embryos and WT littermates. There was no detectable Miner1 protein in the KO MEFs (Fig 2A). However, the abundance of mitoNEET, the related CDGSH family protein localized to the outer mitochondrial membrane, was markedly increased in the KO MEFs. This possibly represents a compensatory upregulation, which might imply similar functions for the CDGSH domains in these family members. ER stress and UPR induction in Miner1 WT and KO MEFs were investigated on multiple levels. There are three major signalling arms of the UPR, which are mediated by the proteins PERK, IRE1 and ATF6 (Oslowski & Urano, 2011). The mRNA levels of the ER stress pathway proteins Bip (an IRE1 target gene) and CHOP (a pro-apoptotic PERK target gene) were increased significantly in Miner1 KO cells, compared with WT controls (Fig 2B); Bip and CHOP protein levels also were increased (Fig 2C). The greater than 3-fold increase in Bip mRNA and the 5.5-fold increase in CHOP mRNA are comparable to or greater than those reported for *Wfs1* deficient islets, MIN6 and INS1 cells (Fonseca et al, 2005; Yamada, 2006). To query activation of the ATF6 arm of the UPR, XBP1 gene expression was analysed, revealing an increase in the Miner1 KO MEFs (Fig 2B). Thus, Miner1 KO cells showed changes in gene and protein expression consistent with activation of the UPR.

Another hallmark of cells undergoing ER stress and the UPR is an expansion of the ER to increase the capacity to process the accumulated unfolded proteins (Zheng et al, 2011). Immunofluorescence staining of WT and KO cells using an antibody to the ER protein calreticulin revealed that the KO cells were larger and that they displayed an increase in the density of the reticular staining pattern throughout the cell (Fig 2D), suggesting ER expansion. Electron microscopy (EM) of the KO cells showed areas of ER with what appeared to be a swollen lumen (Fig 2E). Together, these data demonstrate that MEFs lacking the Miner1 protein do indeed show signs of ER stress/UPR, suggesting this is a common feature in the development of Wolfram Syndrome.

### Miner1 deletion results in dysregulation of Ca^2+^ homeostasis

**Figure 3:**
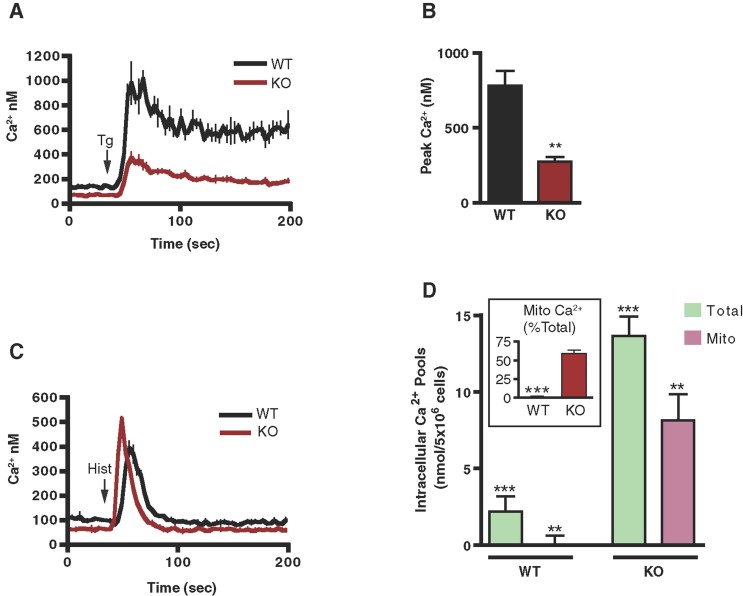
Miner1 deletion results in dysregulation of Ca^2+^ homeostasis Miner1 wildtype (WT) and knockout (KO) MEFs were treated with 2 µM Tg to induce Ca^2+^ release from the ER lumen. Ca^2+^ was measured in the cytoplasm of live cells using Fura2-AM. Mean ± SE of 6 replicates from a representative experiment.Peak Ca^2+^ release (maximum minus basal) in WT and KO MEFs after 2 µM Tg treatment in experiments performed as in (A). (*t*-test, *n* = 3, *p* = 0.0012). Mean ± SE.Ca^2+^ release in WT and KO MEFs via the IP_3_ receptor after treatment with 100 µM histamine (Mean ± SE of 6 replicates from a representative experiment).Quantification and distribution of intracellular Ca^2+^ pools in WT and KO MEFs, assessed using Calcium Green 5N. (ANOVA, *n* = 3) ***KO total > WT total (*p* < 0.0001). **KO mito > WT mito (*p* < 0.0001). Inset shows mitochondrial Ca^2+^ (as a % of total) for each cell type (*t*-test, *n* = 3, *p* = 0.0002). Graphs show Mean ± SE.

ER stress occurs when the demand for ER protein folding exceeds the capacity. Because many ER chaperone proteins are Ca^2+^ dependent, alterations in the Ca^2+^ concentration of the ER lumen can lead to ER stress (Eizirik et al, 2008). Given the ER stress observed in the Miner1 KO MEFs, we used the Ca^2+^ sensitive fluorescent dye Fura-2-AM to investigate whether a lack of Miner1 protein leads to Ca^2+^ dysregulation. There was a lower basal level of cytosolic Ca^2+^ in Miner1 KO cells, and significantly less Ca^2+^ was released from the ER in response to thapsigargin (Tg), an inhibitor of the ER sarco-endoplasmic reticulum Ca^2+^ ATPase (SERCA) (Fig 3A). The peak response (Max signal – basal) was 65% lower in the KO cells relative to the WT (Fig 3B). These data are similar to the changes observed in WFS1 knock down cells (Takei et al, 2006). The inositol triphosphate (IP_3_) receptor is a major ER Ca^2+^ efflux channel. To assess agonist-induced Ca^2+^ release, cells were treated with histamine to stimulate the production of IP_3_ and the opening of IP_3_-dependent Ca^2+^ channels. Interestingly, ER Ca^2+^ release in response to histamine was more profound in the KO than in WT cells (Fig 3C), possibly indicating that the cells lacking Miner1 are primed for ER Ca^2+^ efflux.

In addition to the ER, mitochondria play a dominant role in cellular Ca^2+^ handling. Mitochondria rapidly sequester Ca^2+^ via the mitochondrial Ca^2+^ uniporter. The mitochondrial uptake of ER-released Ca^2+^ is facilitated by the localization of the ER Ca^2+^ efflux channels to sites where the ER and mitochondria physically interact.

To test the model that Ca^2+^ released by the ER due to the absence of Miner1 accumulates in the mitochondria, digitonin-permeabilized Miner1 WT and KO MEFs were analysed fluorimetrically using the Ca^2+^-sensitive dye, Calcium Green 5N and agents that induce the release of Ca^2+^ from the mitochondria (FCCP and antimycin A). Subsequently, the total release of Ca^2+^ from all internal stores was achieved by the addition of alamethicin (Alm), followed by pulses of Ca^2+^ to serve as an internal calibration curve (Supporting Information Fig S3 and Methods). As predicted, there was a dramatic increase in mitochondrial Ca^2+^ content in cells lacking Miner1 (Fig 3D and Supporting Information Fig S3). In fact, the total cellular Ca^2+^ content, determined using Calcium Green and Alm, in the Miner1 KO cells was 6.3-fold greater than in WT cells, due primarily to this increase in mitochondrial load. Even more striking was the alteration in the relative contribution of the separate Ca^2+^ stores to the total pool. In WT cells, the majority of the total Ca^2+^ measured was present in non-mitochondrial pools, whereas in Miner1-deficient cells, the mitochondrial Ca^2+^ pool represented 60% of the total (Fig 3D inset). Our data establish Miner1 as a key determinant in regulating not only ER, but also mitochondrial and total cellular Ca^2+^ homeostasis.

### Miner1 KO MEFs show altered mitochondrial function and ultrastructure

**Figure 4:**
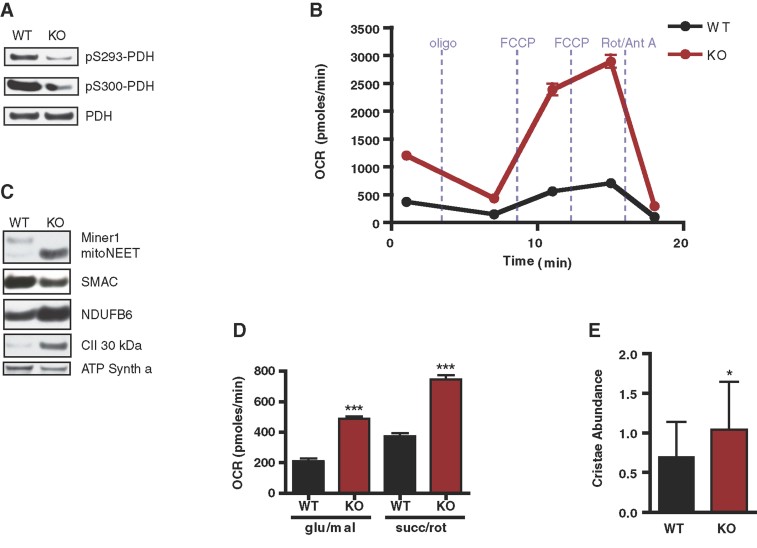
Mitochondria lacking Miner1 have increased respiratory capacity and cristae abundance Source data is available for this figure in the Supporting Information. Western blot analysis of whole cell lysates (20 µg protein) from wildtype (WT) and Miner1 knockout (KO) cells, probed with phospho-specific PDH E1α and total PDH antibodies.Representative Seahorse measurement of OCR (2 × 10^6^ cells) of WT and KO MEFs.Western blot analysis of whole cell lysates from WT and KO cells, loading based on equal cell numbers. Blots were probed with antibodies against various mitochondrial proteins and Miner1. SMAC localizes to the mitochondrial intermembrane space. Inner membrane proteins included ETC components NDUFB6 (Complex I), CII 30 kDa protein (Complex II) and ATP synthase alpha subunit (Complex V).Maximal FCCP-stimulated OCR of mitochondria purified from WT and KO MEFs. Either glutamate/malate or succinate/rotenone were offered as oxidizable substrates (*t*-test, *n* = 4, *p* < 0.0001 for each substrate pair). Mean ± SE.Cristae abundance (cristae area/outer mitochondrial membrane area per mitochondrion) was increased in KO MEFs. Measurements were made from multiple EM micrographs (*t*-test, *n* = 20, *p* = 0.033). Mean ± SE.

The significant increase in mitochondrial Ca^2+^ loading seen in Miner1 KO cells prompted us to investigate various aspects of mitochondrial physiology. Several matrix dehydrogenases are stimulated by Ca^2+^. In particular, the pyruvate dehydrogenase complex (PDH) is regulated by a Ca^2+^-dependent phosphatase that counteracts inhibitory phosphorylation on the PDH E1α subunit. WT and Miner1-deficient cells were analysed by Western blotting with phospho-specific antibodies to two E1α phosphorylation sites on PDH, pSer 293 and pSer 300, whose phosphorylation is inversely correlated with activity (Linn et al, 1969; Rardin et al, 2009). We observed a substantial reduction in Ser 293 and Ser 300 phosphorylation in Miner1 KO cells (Fig 4A), consistent with the observed increase in the Ca^2+^ levels in the mitochondrial matrix (Fig 3D) and possibly substrate flux through PDH.

Immunofluorescence staining for the mitochondrial marker cytochrome c revealed a similar reticular pattern in many WT and Miner1 KO MEFs; however, the KO population had more cells that displayed a punctate mitochondrial pattern (Supporting Information Fig S4A). Given the elevated mitochondrial Ca^2+^ load and punctate morphology, we anticipated that mitochondrial respiratory function might have been compromised. Unexpectedly, when O_2_ consumption rates (OCRs) were measured, Miner1 deficient MEFs displayed a significantly higher OCR relative to WT MEFs, for both FCCP-stimulated maximal rates, as well as basal rates of respiration (Fig 4B). To determine if this reflected an increase in the amount of mitochondria per cell, lysates were prepared from the same number of cells from both KO and WT MEFs and analysed for abundance of mitochondrial proteins. Although we did not observe a general increase in the levels of mitochondrial proteins in Miner1 KO cells, we did detect an increase in several components of the electron transport chain (ETC) (Fig 4C). To eliminate the complications inherent in monitoring whole cell OCR in different cell populations in which mitochondrial mass may differ, we isolated mitochondria from WT and Miner1 KO MEFs and directly assayed O_2_ consumption using the Seahorse XF24 instrument, following our published protocol (Rogers et al, 2011). As detected using whole cells, the OCR of isolated mitochondria from Miner1-deficient MEFs was significantly higher than that of WT cells (Fig 4D).

Despite the enhanced respiratory capacity, the ADP/ATP ratio in the Miner1 KO cells was higher than in controls (Supporting Information Fig S4B), and the DNA synthesis levels in Miner1 KO cells were lower than in WT cells (Supporting Information Fig S4C). The difference in OCR between the basal rate and oligomycin-induced rate represents the amount of oxygen consumption that is utilized for ATP production in the cell (Brand & Nicholls, 2011). Although the rates of respiration after addition of oligomycin were similar in both cell types (indicating a similar level of proton leak), the magnitude of the decrease in response to the ATP synthase inhibitor was much greater in the KO cells (Fig 4B), which suggests enhanced ATP utilization. The increased ADP/ATP ratio is also consistent with an enhanced rate of ATP utilization. Together, these data indicate that the mitochondria of Miner1-deficient cells have an increase in maximal OCR, possibly associated with Ca^2+^-stimulated increase in substrate flux through PDH and the TCA cycle, combined with an increase in ETC components per mitochondria. Furthermore, the bioenergetics data suggest an enhanced energy demand in the Miner1 KO cells.

Given the elevated Ca^2+^ load in the Miner1 KO cell mitochondria, it was surprising that the mitochondria did not appear damaged on a functional level. To analyse further the structural integrity and abundance of the mitochondria, we employed EM and morphometric analysis. Miner1 KO cells did not contain an increased volume of mitochondria per cell (Supporting Information Fig S4D), but the relative surface area of mitochondrial cristae was increased (Fig 4E). Because ETC complexes reside in the cristae of the IMM, the EM data are consistent with the increased abundance of ETC components detected by Western blot and the increased maximal respiratory capacity. In addition, we observed increased numbers of mitochondria containing extensions with either very few or no cristae and small mitochondria devoid of cristae in the Miner1 KO cells (Supporting Information Fig S4E–F), possibly representing damaged mitochondria destined for mitophagy.

Miner1 KO MEFs display higher levels of mitoNEET protein relative to controls. Because mitoNEET is a *bona fide* mitochondrial protein (Wiley et al, 2007a), it is conceivable that the phenotype observed in the Miner1 KO MEFs was the result of mitoNEET overexpression. To investigate this possibility, we generated Miner1 WT and KO MEF cells with a doxycycline-inducible mitoNEET shRNA (or luciferase shRNA controls), as well as Miner1 WT MEF cells stably overexpressing mitoNEET to levels observed in Miner1 KO cells, and analysed them for mitochondrial parameters and ER stress. Reduction of mitoNEET protein in Miner1 KO MEFs had no effect on Bip or CHOP mRNA levels, phosphorylation of PDH, nor mitochondrial morphology (Supporting Information Fig S5A–D). Likewise, overexpression of mitoNEET in Miner1 WT cells did not result in significant changes in Bip or CHOP mRNA levels, PDH phosphorylation or oxygen consumption rates (Supporting Information Fig S6A–D). This data does not support a causative role for elevated mitoNEET expression in the development of the observed phenotype in the Miner1 KO MEFs.

It would appear that the loss of Miner1 at the ER creates a situation that impinges on the mitochondria, likely as a function of ER-mitochondrial communication and altered Ca^2+^ homeostasis.

### Lack of Miner1 leads to a more oxidized intracellular milieu

The only defined functional domain in the Miner1 protein is the CDGSH domain, which binds a redox-active 2Fe-2S cluster, leading to the speculation that Miner1 is involved in redox regulation. To determine if Miner1 can affect the redox status of the cell, we monitored a variety of redox-sensitive processes.

**Figure 5:**
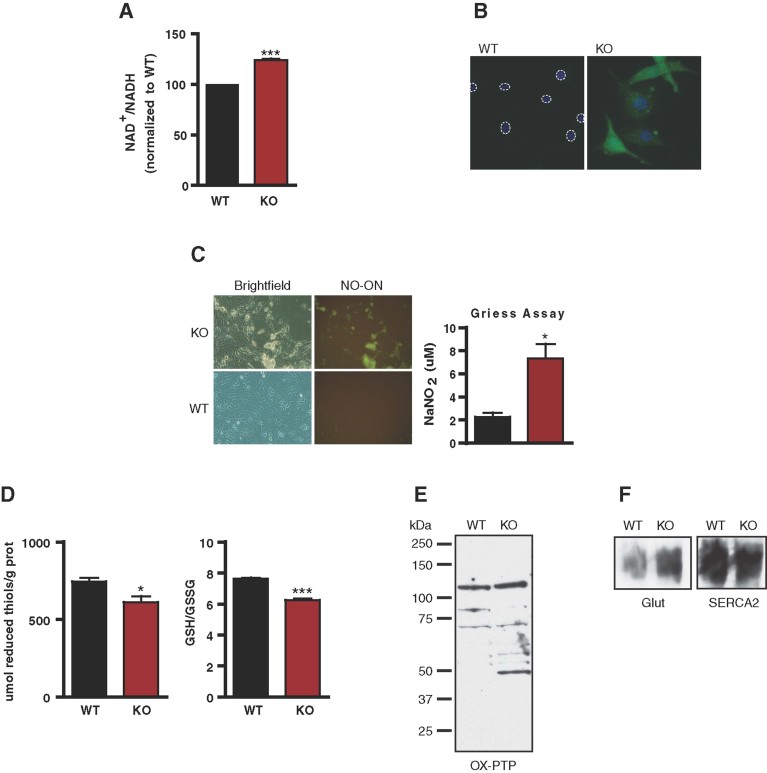
Shift towards oxidized environment in Miner1 KO cells NAD^+^/NADH ratios (normalized to WT) in wildtype (WT) and Miner1 knockout (KO) MEF lysates (*t*-test, *n* = 4, *p* < 0.0001). Mean ± SE.Live cell imaging at 20X magnification of WT and KO MEFs after labelling with the Image-iT LIVE Green ROS indicator and co-staining nuclei with DAPI (blue). Intensity of green fluorescence is proportional to amount of ROS in cell. Dashed circles mark nuclei in WT.Brightfield (left panels) and fluorescent (right panels) images of WT and KO MEFs at 10× magnification after labelling with NO–ON. Intensity of green fluorescence is proportional to amount of NO in cell. Images were captured at identical camera settings for B and C. Graph at right reports Griess Assay quantitation of NO_2_ in WT and KO MEF cell culture media (*t*-test, *n* = 4, *p* = 0.008). Mean ± SE.Left panel is FluoroThiol assays with WT and KO MEF lysates to quantitate total reduced thiols per g of protein (*t*-test, *n* = 3, *p* = 0.0393). Right panel is graph of GSH/GSSG ratios in WT and KO cells (*t*-test, *n* = 3, *p* = 0.0005). Mean ± SE.Western blot analysis of WT and KO WCL with antibody to oxidized active site cysteine of PTPs.Non-reducing SDS-PAGE and Western blots of SERCA2 immunoprecipitates from WT and KO whole cell lysates. PVDF membranes initially blotted with an antibody to glutathione adducts on proteins (Glut), then stripped and probed for total SERCA2.

Many redox reactions in the cell are coupled to the NAD^+^/NADH redox pair. There was an increase in the NAD^+^/NADH ratio (Fig 5A), indicative of a more oxidized milieu in the KO cells. To detect levels of cellular ROS production in Miner1 WT and KO MEFs, we utilized the cell-permeable indicator carboxy-H_2_DCFDA. The fluorescence in WT cells was faint; however, Miner1 KO cells exhibited a robust fluorescence signal (Fig 5B), suggesting increased ROS production in the KO cells. NO levels were imaged in live cells loaded with the NO-specific fluorescent dye NO-ON. Miner1 KO MEFs exhibited markedly increased fluorescence compared with controls, indicating substantially increased NO production (Fig 5C). We also used the Griess assay to measure nitrite concentrations in the cell culture media. In agreement with the results of live fluorescence imaging, the Griess assay also suggested an increase in NO production in Miner1-deficient MEFs (Fig 5C). Thus, Miner1 KO MEFs exhibit multiple signs of reactive oxygen and nitrogen-mediated stress.

Many proteins critical to ER-mitochondrial signalling, such as Ca^2+^ channels and phosphatases, have redox sensitive cysteines, whose oxidation state can regulate activity (Karisch et al, 2011; Sammels et al, 2010; Tonks, 2006). In addition, the oxidation status of the cysteine-containing tripeptide glutathione, a major anti-oxidant in the cell, reflects the level of oxidative stress (Biswas et al, 2006). To probe the general oxidation state of total thiols in the cell, lysates from WT and KO MEFs were assayed using the redox-sensitive fluorescent dye, Fluorothiol. Miner1 KO MEFs displayed a decrease in total reduced thiols (Fig 5D), reflecting a shift towards a more oxidized state of protein thiols. Direct measurement of oxidized glutathione (GSH/GSSG) (after deproteination) also confirmed the more oxidized status of KO MEFs (Fig 5D).

Members of the protein tyrosine phosphatase (PTP) CX_5_R family play critical roles in signalling throughout the cell, including in the ER and mitochondria. Dysfunction of several members of this family have been implicated in both diabetes and aging (Koren & Fantus, 2007; Wei et al, 2011). PTPs possess an essential redox-sensitive cysteine in their active site that, when oxidized, results in enzyme inactivation. This oxidation can be reversible (sulphenic acid) or irreversible (sulphinic and sulphonic acid). Because the oxidative shift in the Miner1 KO cells was subtle, we specifically examined the oxidation state of CX_5_R PTPs to determine if the oxidative stress in the KO cells would be sufficient to affect the activity of this important class of signalling molecules. WT and Miner1 KO MEFs were lysed in degassed buffer containing reducing agents, cleared of insoluble material and analysed by Western blotting with an antibody specific to the sulphonic acid oxidation state of the catalytic cysteine of many PTPs (OX-PTP). There was an increase in the number of reactive bands in the Miner1 KO lysates. We also investigated the oxidation state of specific PTPs using a recently developed mass spectrometry technique (Karisch et al, 2011), which also revealed an increase in PTP oxidation (expressed as % oxidized, Supporting Information Table S1). The spectrum of PTPs that were oxidized suggests that a number of different signalling networks are likely to be affected in Miner1 deficient cells.

The primary proteins mediating both ER Ca^2+^ influx (SERCA) and efflux (IP_3_R and the ryanodine receptor, RYR) channels contain redox-sensitive cysteines that affect channel activity (Andersson et al, 2011; Sammels et al, 2010; Tong et al, 2010). In addition to the cysteine oxidation states mentioned above, redox-active cysteines in SERCA can be oxidatively modified by adduction with glutathione (glutathionylated) in response to elevated NO, which alters its activity. Oxidation of the IP_3_ receptor and RyR channels are reported to increase their channel activity, resulting in Ca^2+^ leak from the ER. SERCA2 immunoprecipitation, followed by anti-glutathione Western blotting, revealed increased levels of glutathione adducts in the KO cells (Fig 5F). These data suggest that cells lacking Miner1 display multiple signs of oxidative stress.

### Anti-oxidant treatment reverses stresses induced by lack of Miner1

**Figure 6:**
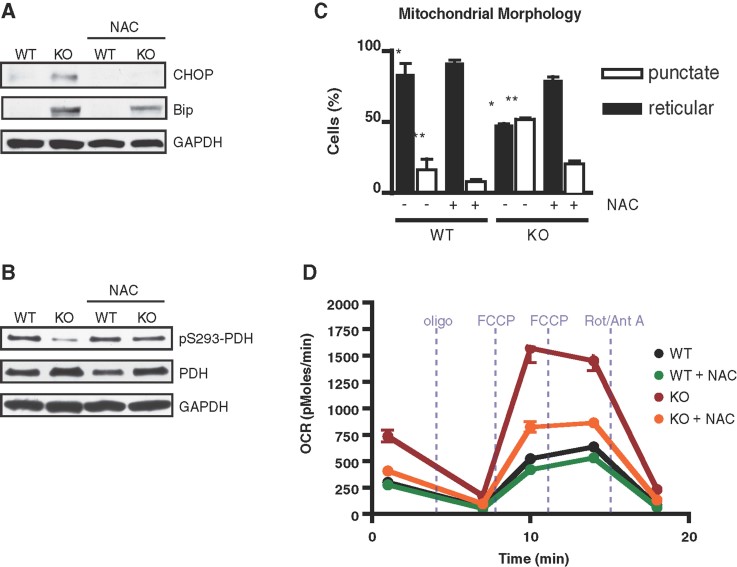
Anti-oxidant treatment mitigates stresses induced by Miner1 deficiency Source data is available for this figure in the Supporting Information. **A,B.** Western blot analysis of whole cell lysates from wildtype (WT) and Miner1 knockout (KO) MEFs with and without 5 mM NAC for 48 h (15 µg protein).**C.** Quantitation of anti-cytochrome c immunofluorescent analysis of reticular or punctate mitochondrial morphology. WT and KO MEFs were untreated or treated with 5 mM NAC for 48 h. (*n* = 3 experiments. 80–200 cells scored per experiment. Reticular and punctate morphology % compared by ANOVA (asterisks denote *p* < 0.0001, * WT reticular > KO reticular, **WT punctate < KO punctate, NAC treated WT & KO samples do not differ from WT untreated).**D.** Representative Seahorse experiment of OCR (2 × 10^6^ cells) of WT and KO MEFs that were untreated or treated with 5 mM NAC for 48 h prior to the experiment.

As lack of Miner1 results in oxidative stress, we asked whether anti-oxidant treatment might reverse the anomalies observed in Miner1 KO MEFs. To test this possibility, WT and Miner1 KO MEFs were treated for 48 h with anti-oxidants and evaluated for ER stress responses and mitochondrial function. Treatment with 5 mM NAC for 48 h resulted in an amelioration of many of the anomalies detected in the Miner1 KO MEFs. There was virtually none of the pro-apoptotic CHOP protein detected in NAC-treated KO MEFs and a reduction in the amount of Bip protein (Fig 6A), suggesting that the ER stress resulted from an oxidative stress.

Our data are consistent with a model of oxidative stress inducing Ca^2+^ leak from the ER, which is sequestered into the mitochondria, thereby stimulating dephosphorylation of PDH. We used PDH phosphorylation as a surrogate monitor of mitochondrial Ca^2+^ load. While pS293 was substantially reduced relative to WT in the untreated KO cells, pS293-PDH was returned to the levels observed in the WT cells after NAC treatment (Fig 6B). We also examined mitochondrial homeostasis at the level of morphology and function. NAC treatment resulted in a return to a reticular morphology in the KO mitochondria (Fig 6C). After NAC treatment, the increased respiration rates observed in the KO cells were restored to levels virtually the same as those in WT cells (Fig 6D). These experiments strongly suggest that oxidative stress lies at the root of the ER and mitochondrial changes caused by Miner1 deficiency, and importantly that sulphydryl anti-oxidant treatment could be a rational therapeutic approach for treating this syndrome.

### Re-expression of Miner1 partially rescues the Miner1^(−/−)^ MEF phenotype

**Figure 7:**
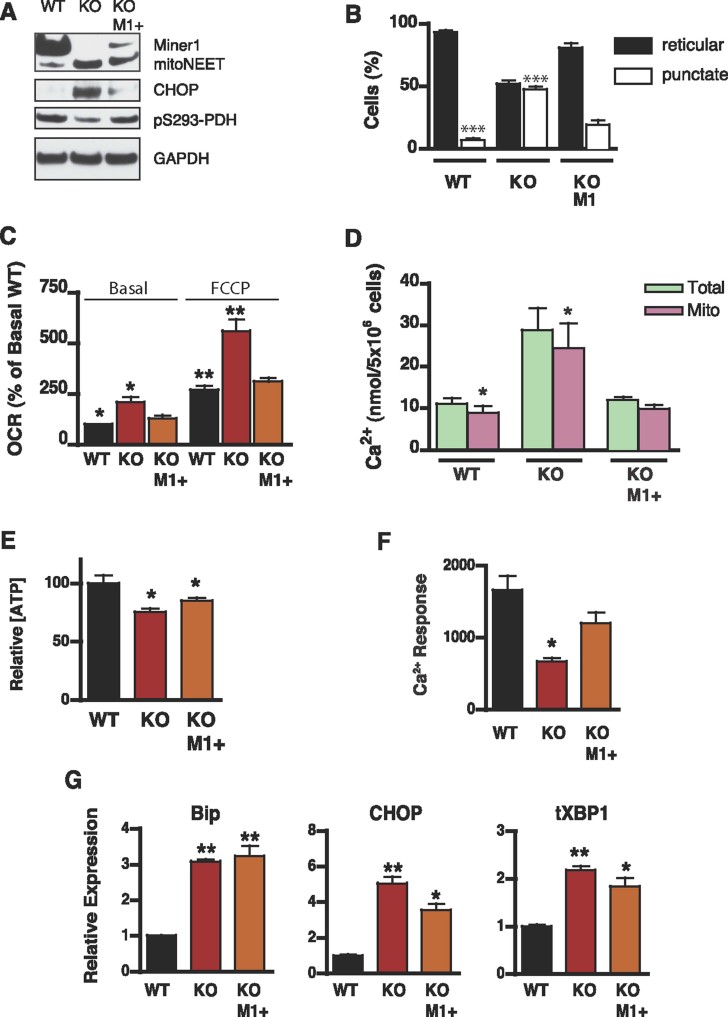
Characterization of Miner1 re-expression in KO cells Miner1 wildtype (WT) and Miner1 knockout (KO) MEF cell lines, including KO (Miner1^−/−^) MEFs exogenously re-expressing Miner1 (KO-M1^+^). Western blot analysis of cell extracts (30 µg protein).Characterization of mitochondrial morphology, as in Fig 6C. (ANOVA, *n* = 3 experiments. 80–200 cells scored per experiment (asterisk denotes *p* < 0.0001, WT punctate < KO punctate; KO/M1 punctate does not differ from WT).Oxygen consumption rates (OCR) were measured in a Seahorse XF24. Basal and maximal FCCP-stimulated rates relative to WT basal are shown. (*t*-test, *n* = 6, asterisk denotes *p* < 0.0001, *WT basal < KO basal, **WT FCCP < KO FCCP; KO/M1 values do not differ from WT).Mitochondrial Ca^2+^ measured using Calcium Green 5N fluorescence. Permeabilized cells were assayed for total and mitochondrial Ca^2+^ as in Fig 3D. Values reported are raw Ca^2+^ measurements. Average contaminating Ca^2+^ was 9.52 nmol. (ANOVA, WT & KO, *n* = 5; KO-M1^+^, *n* = 3; *WT mito < KO mito, *p* = 0.035, KO/M1 values do not differ from WT).ATP levels relative to cell number (cps/RFU). KO and KO-M1^+^ relative to WT (ANOVA, *n* = 3). WT > KO (*p* = 0.0275), WT > KO-M1^+^ (*p* = 0.0312).ER Ca^2+^ release after treatment with 2 µM Tg, expressed as peak responses (Ca^2+^ – basal Ca^2+^) (ANOVA, *n* = 3) WT > KO (*p* = 0.0004), KO-M1^+^ > KO (*p* = 0.0087).qRT-PCR analysis of ER stress markers. Bip, CHOP, and total XBP1 expression were monitored (ANOVA, *n* = 3). Bip: **WT < KO & KO-M1^+^ (*p* < 0.0001); CHOP: **WT < KO (*p* = 0.0005), WT < KO-M1^+^ (*p* = 0.0023), *KO-M1^+^ < KO (*p* = 0.0486); tXBP1: **WT < KO (*p* = 0.0002), *WT < KO-M1^+^ (*p* = 0.0085).

To support that the observed ER and mitochondrial changes in the Miner1 KO cells was due to lack of Miner1 protein, we re-expressed Miner1 protein in the Miner1^(−/−)^ cells and probed for reversal of the aberrant phenotype. Miner1 cDNA was introduced into the KO cells using lentivirus transduction and neomycin-resistant pools of cells (referred to as KO-M1^+^ cells) were used to analyse ER and mitochondrial parameters. The level of Miner1 exogenous expression obtained was substantially less than in WT cells; however, it was sufficient to rescue the mitochondrial parameters measured. PDH phosphorylation in the KO-M1^+^ cells was similar to that in WT cells, as were mitochondrial morphology, oxygen consumption rates and mitochondrial Ca^2+^ load, measured by Calcium Green fluorescence as in Fig 3D (Fig 7A–D). Although the mitochondrial homeostasis was normalized, relative ATP levels in the KO-M1^+^ cells remained lower than in WT cells (Fig 7E). There was also less than complete rescue of ER homeostasis, presumably due to the incomplete replenishment of Miner1 protein levels. The ER Ca^2+^ content of the KO-M1^+^ rescue cells was significantly elevated compared to KO; however, the level was not as great as that in WT cells, although not statistically different from WT (Fig 7F). Indicators of ER stress revealed a partial rescue, consistent with modest rescue of Miner1 protein levels. There was a reduction of CHOP protein in the KO-M1^+^ cells, but mRNA levels of Bip, CHOP and tXBP1 remained elevated (Fig 7A,G).

## DISCUSSION

Wolfram Syndrome was long thought to be a disease of reduced ATP supply, suggesting mitochondrial dysfunction. This view was based in part on bioenergetic deficits detected in patients with this disease. In addition, the tissues affected tend to be those with high energetic demands (Bundey et al, 1992). The data presented here suggest that the metabolic dysfunction in WFS2 arises not from a primary mitochondrial defect, but as an indirect consequence of altered Ca^2+^ homeostasis at the ER caused by an alteration of the sulphydryl redox status. We and others have previously demonstrated that a recombinant CDGSH domain protein expressed in *Escherichia coli* is redox active *in vitro* (Wiley et al, 2007b). However, data supporting a redox role for Miner1 in cells has been lacking. In the current study, we have established the ability of Miner1 to influence the general redox status in the cell. Miner1 deficient cells displayed changes in NAD^+^/NADH ratio, NO production, GSH/GSSG ratio, protein glutathionylation and oxidation of sulphydryl groups, including those in CX_5_R phosphatases. We found that Miner1 KO MEFs have a more oxidized cytoplasmic environment and show signs of oxidative stress. The orientation of the Miner1 CDGSH domain on the cytoplasmic face of the ER is consistent with a role in regulating cytoplasmic redox status, and Miner1 KO cells exhibit a general increase in sulphydryl oxidation. The catalytic Cys of the PTP CX_5_R family of phosphatases is exquisitely sensitive to sulphydryl oxidation, resulting in inactivation of the enzymes. Thus, these phosphatases can be viewed as protein sentinels reflecting the cellular redox status. The increase in oxidation of catalytic Cys in phosphatases detected in the mutant MEFs is likely to have wide-ranging effects on signalling by reversible phosphorylation.

In addition to redox regulation of phosphatase activity, the oxidative environment of the Miner1 KO MEFs could lead to oxidation of regulatory cysteines on ER Ca^2+^ transporters and channels. Oxidative modifications of ER Ca^2+^ transporters have been shown to affect transport activity and lead to a decrease in Ca^2+^ in the ER lumen, resulting from alteration of the activity of SERCA and increased leak via RyR or IP_3_ receptors (Sammels et al, 2010). We observed an increase in SERCA2 glutathionylation in Miner1 deficient cells, suggesting that the decrease in ER Ca^2+^ levels may be caused by changes in SERCA2 transport activity. Oxidation of IP_3_ receptors may also contribute to the Ca^2+^ dysregulation observed in the Miner1 KO cells. As a redox-active protein anchored to the ER and enriched in MAMs, Miner1 is in a prime location to directly interact with these transporters and keep them in a reduced state.

Because many of the folding chaperones in the ER are Ca^2+^-dependent, decreased ER Ca^2+^ content has been linked to ER stress and induction of the UPR. We found that MEFs derived from Miner1 KO mice also display the hallmark signs of ER stress/UPR. Miner1-deficient MEFs exhibit elevated levels of the folding chaperone Bip and the pro-apoptotic protein CHOP, as well as an expanded ER network.

Enhanced Ca^2+^ leak from the ER as a result of oxidative protein modification can explain the increased mitochondrial Ca^2+^ load that we observed in Miner1 KO cells and the increase in dephosphorylated PDH. Oxidation of RyR Ca^2+^ channels have been associated with increased Ca^2+^ leak from the ER, increased mitochondrial Ca^2+^ loading and increased muscle wasting in mice (Andersson et al, 2011). Sarcopenia was also observed in the Miner1 KO mice (Chen et al, 2009). Enhanced ER Ca^2+^ leak would also predict the elevated ADP/ATP ratio and the enhanced rate of ATP utilization we observed in the rates of endogenous O_2_ consumption (Norris et al, 2010). These findings, along with a decreased growth rate, are consistent with an underlying mechanism of enhanced futile Ca^2+^ cycling.

There is precedent for altered mitochondrial Ca^2+^ loading, morphology and function caused by changes in ER protein function. Expression of a truncated isoform of SERCA1 results in ER Ca^2+^ leak, mitochondrial Ca^2+^ loading, changes in mitochondrial morphology and increased ER-mitochondrial contacts (Chami et al, 2008). Similarly, mutations in presenilin2, an intramembrane protease that is localized to the ER and that is linked to the onset of Alzheimer's Disease, influences ER-mitochondrial Ca^2+^ cross-talk and physical contacts (Zampese et al, 2011). Consistent with these paradigms of ER-mitochondrial Ca^2+^ dysregulation, we found that Miner1 KO MEFs have altered mitochondrial structure. Surprisingly, mitochondria isolated from Miner1 KO MEFs demonstrated much higher respiratory capacity than WT mitochondria, in contrast to the mitochondria isolated from muscle of Miner1 KO mice, which showed virtually no ADP-stimulated respiration (Chen et al, 2009). It is possible that immortalization of the MEFs in culture promotes tolerance and adaptation to the mitochondrial Ca^2+^ loading and elevated ROS, perhaps via a compensatory increase in cristae area and expression of ETC components. We predict that an electrically excitable cell type, such as a neuron or myocyte, would not tolerate this form of Ca^2+^ dysregulation and would be more likely to suffer from mitochondrial dysfunction. The phenotype of a recently reported second mouse model of Miner1 deficiency is significantly milder than the mouse established by Chen and colleagues (Chen et al, 2009) (the source of our fibroblasts) and lacks features present in the human disease (Chang et al, 2012). However, the increase in cristae density that we observe in the fibroblasts is recapitulated in skeletal muscle myocytes of the mouse established by Chang and coworkers (Chang et al, 2012).

Wolfram Syndrome patients present with diabetes mellitus and diabetes insipidus, which result from compromised production of insulin by the pancreas and of vasopressin by the pituitary, respectively. Both of these are secretory organs that place a tremendous demand on the ER for protein folding. The chronic ER stress created by Miner1 deletion is likely to overwhelm the protein folding and secretory capacity of these organs in the short term, and ultimately lead to organ failure. The blindness and deafness observed in Wolfram Syndrome is of neuronal origin, and the chronic oxidative stress, mitochondrial Ca^2+^ loading and lower ATP levels that we observed could lead to chronic dysfunction or death of electrically excitable cells. The sarcopenia observed in Miner1 KO mice could reflect low ER Ca^2+^ levels in muscle. Lastly, oxidative stress has been correlated with both aging and the diabetic state (Giacco & Brownlee, 2010; Haigis & Yankner, 2010). Thus, our cell culture model has enabled us to develop a mechanistic model for the manifestations of Wolfram Syndrome observed in human patients and knockout mice (Supporting Information Fig S7).

Our studies form the basis of a new paradigm for the function of Miner1 and the etiology of Wolfram Syndrome. We conclude that Miner1 is a redox protein that resides in the ER and that regulates the UPR and mitochondrial function. Consequently, our data suggests that the defects observed in Miner1 MEFs stem from oxidative stress, which can be mitigated by treatment with anti-oxidants. Indeed, application of the sulphydryl anti-oxidant NAC reversed many of the mitochondrial and ER stress-related anomalies observed in the Miner1 KO cells, hence anti-oxidant treatment represents a potentially promising therapeutic strategy for treating this currently incurable disease.

## MATERIALS AND METHODS

Further details can be found in Supporting Information.

### Cell culture and reagents

Miner1 (Cisd2) WT and KO MEFs were immortalized using a retroviral vector expressing T antigen. Construction of cell lines to modulate levels of Miner1 and mitoNEET are described in detail in Supporting Information Methods. Transient transfections of HEK293T and COS7 cells were performed using Fugene (Roche). Cells were harvested for microsomal isolation or fixed for immunofluorescent staining 24–48 h post-transfection. Unless stated, all chemicals were from Sigma–Aldrich.

### Tissue/cell fractionation and manipulation

Mitochondria, MAM, ER and cytosolic (soluble) fractions were prepared from rat livers and analysed by Western blot as described (Wieckowski et al, 2009). Microsomal ER fractions were prepared from fresh rat livers and subjected to high salt and high pH washes, followed by trypsinolysis as previously described (Wiley et al, 2007a).

### Western blots and immunofluorescence analysis

In general, equivalent amounts of protein were loaded for SDS-PAGE and Western blotting. MitoTracker Red and immunostaining was performed as described (Wiley et al, 2007a). Cells were imaged with a Leica DMR microscope and a Hamamatsu camera. Images were processed with OpenLab Software.

### Real-time PCR and statistical analyses

mRNA was isolated using the RNeasy miniprep kit (Qiagen). cDNA synthesis was performed using the iScript kit (Bio-Rad), and qRT-PCR performed using Syber Green PCR Master Mix (ABI). See Supporting Information for primer sequences and further details. Students *t*-tests and ANOVAs with Tukey post-tests were performed using Prism Graph Pad Software. *n*-values represent number of separate experiments. Values of *p* < 0.05 (*) were considered statistically significant [*p* < 0.01 (**); *p* < 0.001 (***)]. All data are presented as mean ± standard error of the mean (S.E.M.) unless otherwise specified.

### Ca^2+^ assays

ER Ca^2+^ stores were quantified following 2 µM Tg-induced ER Ca^2+^ release using Fura2-AM (Invitrogen) and a FLEX-Station (Molecular Devices). ER Ca^2+^ release via the IP_3_ receptor was triggered by treatment with 100 µM histamine. The Ca^2+^ content of mitochondria and other intracellular Ca^2+^ stores was assessed in digitonin-permeablized cells using Calcium Green 5N (Invitrogen) with cells in suspension in a cuvette using the LS-50B spectrofluorometer (Perkin-Elmer). See Supporting Information Fig S3 and Methods for further details.

### Mitochondrial respiration, ATP, cell proliferation and redox measurements

The Seahorse XF24 Extracellular Flux Analyzer was used to determine OCR as a measure of mitochondrial respiratory function in whole cells and isolated mitochondria. OCR replicates varied from 3 to 6 per experiment. ADP/ATP ratios were determined using the fluorescent ApoSensor ADP/ATP Ratio kit (BioVision). Cell proliferation was assessed by EdU incorporation into DNA using the Click-iT EdU Imaging kit (Invitrogen). Relative ATP levels were also assessed using CellTiter Glo (Promega). NAD^+^/NADH ratios were measured using an NAD/NADH Assay Kit (Abcam). ROS was imaged in live cells using the Image-iT LIVE Green ROS detection kit (carboxy-H_2_DCFDA) (Molecular Probes). Nitric oxide (NO) was imaged using the NO-specific fluorescent probe (NO–ON) (Strem Chemicals). Released nitrite, a stable oxidation product of NO, was measured using the Griess Assay; media was collected from WT and KO MEFs 2 days post-confluence and assayed. Total reduced thiols were measured using the Fluoro Thiol Detection Kit (Cell Technology, Inc.). GSH/GSSG ratios were determined using the Glutathione Assay kit (Cayman Chemical); both were normalized to protein concentration. All assays were performed following the manufacturers' instructions. For oxidized PTP blots, cell lysates were analysed by SDS-PAGE with an antibody recognizing the sulphonic acid oxidation state of the active site cysteine (R&D Systems). The oxidation state of the catalytic Cys in various PTPs was measured by mass spectrometry as described (Karisch et al, 2011). See Supporting Information Methods for further details.

**The paper explained**

PROBLEM:

Wolfram Syndrome (DIDMOAD) is an incurable disease characterized by a range of endocrine and neurological symptoms, including diabetes insipidus, diabetes mellitus, blindness due to optic atrophy and sensorineural deafness. Mutations in the genes encoding Wolframin (an endoplasmic reticulum protein involved in ion channel activity and the unfolded protein response) or Miner1 (a 2Fe-2S cluster-containing protein) lead to the development of DIDMOAD. Full understanding of DIDMOAD is hindered by limited insight into the role of Miner1 in the cell.

RESULTS:

We used fibroblasts immortalized from WT and Miner1 KO mouse embryos to elucidate the cellular processes impacted by Miner1. Deletion of Miner1 results in a more oxidized intracellular milieu, and this is associated with ER stress and the unfolded protein response, profound ER and mitochondrial Ca^2+^ dysregulation, and altered mitochondrial structure and function. Importantly, treatment with the sulphydryl anti-oxidant *N*-acetyl cysteine ameliorates many of the mitochondrial and ER-stress related anomalies observed in the Miner1 KO cells.

IMPACT:

Our studies form the basis of a paradigm for the function of Miner1 and provide novel mechanistic insights into the underlying pathology of DIDMOAD. These experiments strongly suggest that oxidative stress lies at the root of the ER and mitochondrial changes caused by Miner1 deficiency, and importantly that sulphydryl anti-oxidant treatment could be a rational therapeutic approach for treating this syndrome.

### EM and morphometric analysis

Cell growth, preparation and EM analysis were as previously described; ImageJ (NIH) was used to perform morphometric analysis of mitochondrial volume and cristae/OMM surface area as previously described (Zhang et al, 2011).

## For more information

on Miner1 (CISD2), see http://omim.org/entry/611507

on Wolfram Syndrome, see http://ghr.nlm.nih.gov/condition/wolfram-syndrome; on Wolfram Syndrome 2, see http://omim.org/entry/604928, and Wolfram Syndrome 1, see http://omim.org/entry/222300

on the clinical applications of N-acetylcysteine, see Millea, PJ (2009) *Am Fam Physician.* 80(3):265–269, http://www.aafp.org/afp/2009/0801/p265.html

## Supplementary material

As a service to our authors and readers, this journal provides supporting information supplied by the authors. Such materials are peer reviewed and may be re-organized for online delivery, but are not copy-edited or typeset. Technical support issues arising from supporting information (other than missing files) should be addressed to the authors.
