# Supplementary material for: Wolfram Syndrome protein, Miner1, regulates sulphydryl redox status, the unfolded protein response, and Ca2+ homeostasis
Source: EMBO Mol Med. 2013 May 24;5(6):904–18. doi: 10.1002/emmm.201201429 (PMC3779451; doi:10.1002/emmm.201201429)
Supplement: Supplementary file 1 [file emmm0005-0904-SD1.pdf]

EMBO Molecular Medicine

Wolfram Syndrome protein, Miner1, regulates sulfhydryl redox status, the unfolded protein response and Ca2+ homeostasis

Sandra E. Wiley, Alexander Y. Andreyev, Ajit S. Divakaruni, Robert Karisch, Guy Perkins, Estelle A. Wall, Peter van der Geer, Yi-Fan Chen, Ting-Fen Tsai, Melvin I. Simon, Benjamin G. Neel, Jack E. Dixon, Anne N. Murphy

Corresponding author: Anne Murphy, University of California, San Diego, USA

Review timeline:

Submission date:	01 April 2012
Editorial Decision:	29 April 2012
Revision received:	01 August 2012
Editorial Decision:	06 September 2012
Revision received:	04 February 2013
Editorial Decision:	08 March 2013
Revision received:	26 March 2013
Accepted:	27 March 2013

Transaction Report:

(Note: With the exception of the correction of typographical or spelling errors that could be a source of ambiguity, letters and reports are not edited. The original formatting of letters and referee reports may not be reflected in this compilation.)

Editors: Anneke Funk / Céline Carret

1st Editorial Decision 29 April 2012

Thank you for the submission of your manuscript to EMBO Molecular Medicine. We have now heard back from the three referees whom we asked to evaluate your manuscript. Although the referees find the study to be of potential interest, they also raise a number of concerns that should be addressed in a major revision of the work.

As you will see, the referees find the study novel and interesting. However, they do suggest a certain number of additional experiments to strengthen the conclusions that I would encourage you to add.

Of particular relevance, a causal evidence for a direct role of Miner1 in the redox status of the cell should be provided as highlighted by Referee #1 and #2. These two reviewers also suggest performing a MitoNEET over-expression experiment to biologically address the role of MitoNEET. Referee #1 also suggests providing in vivo data to increase the physiological relevance. Finally a more thorough characterization of ER and mitochondria calcium homeostasis would improve the data.

Although we realize that all the requirements of Referee #2 might not all be necessary, I would appreciate if you could try to address as much as you can but in any case please reply to all reviewer's comments in writing.

Given the balance of these evaluations, we would like to invite a revision of your manuscript addressing the issues that have been raised within the space and time constraints outlined below. Please note that it is EMBO Molecular Medicine policy to allow only a single round of revision and that, as acceptance or rejection of the manuscript will depend on another round of review, your responses should be as complete as possible.

Revised manuscripts should be submitted within three months of a request for revision; they will otherwise be treated as new submissions, except under exceptional circumstances in which a short extension is obtained from the editor. Also, the length of the revised manuscript may not exceed 60,000 characters (including spaces) and, including figures, the paper must ultimately fit onto optimally ten pages of the journal. You may consider including any peripheral data (but not methods in their entirety) in the form of Supplementary information.

***** Reviewer's comments *****

Referee #1:

In this study the authors show that Miner1-deficiency in MEF cells result in increased UPR markers, defects in mitochondrial respiration and increase in mitochondrial Ca++ load. The investigators also provide evidence supporting that Miner1-deficiency results in increased ROS production and creates an oxidized milieu as indicated by an increase in oxidized NAD+ and Gluthatione, therefore suggesting a role for direct influence of Miner1 in cellular redox status. The authors relate the increased Ca++ to an oxidative stress induced Ca++ leakage model, which they produce evidence to support this model with treatment of Miner1-deficient cells with NAC. This antioxidant treatment reduces CHOP and Bip levels and rescues the mitochondria phenotype in Miner1 deficient cells. Overall, the findings in this paper are extremely interesting. The results are novel and important and the paper is written well. Some aspects of the study would benefit from further work especially in generating a more thorough characterization of ER and mitochondria calcium homeostasis and a better morphological and/or cell biological analysis following antioxidant treatment. It would also be extremely useful to attempt to demonstrate MitoNEET up regulation or generate another line of more direct evidence to link Miner1-deficiency to the observed phenotype and exclude any potential contributions from MitoNEET overexpression in the experimental system used in the study. Otherwise, this is a high quality study with important information

Specific points for consideration:

1) The main evidence provided in supporting oxidative stress being upstream of calcium leak from ER is the NAC treatment experiments provided in Figure 6, in which no calcium biology was presented. I believe this work can be significantly strengthened if the authors can provide a more thorough characterization of ER and mitochondria calcium homeostasis and their morphologies post-NAC treatment. It is important to establish that NAC treatment does reduce calcium leakage from ER and thus restore ER and calcium mitochondria homeostasis. It has also been previously shown that Ero1a can interact with IP3R to regulate calcium transport from ER to mitochondria. It may be productive to explore whether this well established pathway is involved in the observations presented here. In addition, the seahorse experiment with isolated mitochondria from Miner1-deficient cells (Figure 4D) exhibits higher OCR than those from WT cells, suggesting that the mitochondria function maybe independent of ER calcium release. Can the authors elaborate on the possible mechanism?

2) The authors attribute all the phenotypes of Miner1-deficient cells to Miner1 depletion in the cell. As shown in Figure 1, 2 and 4, a major consequence of Miner1 depletion is MitoNEET upregulation. As MitoNEET is localized to mitochondria and MAM, could it be possible that the observed phenotypes may be influenced by this mechanism? The authors may consider performing a MitoNEET overexpression experiment to support or refute this possibility.

3) Rescue experiments with Miner1 re-introduction back into Miner1-/- cells would provide strong causal evidence for direct involvement of Miner1 in general redox status of the cell. Also a Miner1 mutant protein lacking (or mutated) CDGSH domain would further strengthen the argument for Miner1's role in regulating general redox status of the cell.

4) In vivo studies involving Miner1-deficient models treated with NAC (or any other antioxidant) would strengthen the core findings of the study.

Minor points:

1) Figure 2D, the authors used calreticulin as a marker of ER and ER expansion. ER expansion was not noticeable on the EM level, just swelling. Is it possible that, under such severe ER stress condition (swollen ER), calreticulin is not exclusively restricted to the ER? Could this be tested?

2) The authors showed that Bip level is increased as hallmark of ER stress, what happens to the level of calreticulin, XBP1 splicing or other UPR indicators?

3) Page 13, line#4 from bottom, "suggesting an enhanced ATP utilization", the data may also support enhanced ATP production instead. It is the combination of "enhanced ATP production" but "increased "ADP/ATP ratio" which would support ATP utilization is also enhanced.

4) Page 16, line 9, "Because the oxidative shift in the Miner1 KO cells was subtle...". Why do the authors thing these are subtle changes? Data presented in Figure 5B &C seem quite clear-cut to this reviewer. Can the data be quantified?

5) Can Authors also provide the absolute values of each nucleotide measured? Since the ratio of oxidized to/reduced nucleotides is critical and that oxidation is also a function of reduced pool availability; it would be important to see whether the reduced nucleotides are comparable in both cell lines. Could authors also check NAPP+/NADPH levels as there could be hydride transfers between these two species of dinucleotides.

6) In multiple Figures, the author showed Bip is absent in WT cells. Bip is one of the most abundant proteins in the ER regardless of the status of ER and presence of stress.

7) As NAC treatment could have secondary/off target effects it would be highly valuable to try a different ROS scavenger to strengthen the argument in support of oxidative stress model? Alternatively a genetic model of antioxidant gene over expression in these cells (e.g. Glutathione reductase) could also be tried.

Referee #2:

In this paper authors investigate the subcellular localization of Miner1, and its role in cellular redox state. They claim that the enhanced oxidative intracellular environment of Miner1-/- cells induces general changes in the sulphydryl redox state of the cell. As consequence, ER Ca2+ transporters show altered activity that results in decreased calcium storage in ER and increased calcium load in mitochondria. They further describe bioenergetic and morphological mitochondrial alteration in Miner1-/- MEFs, and accumulation of ROS. In general, even if Miner1 localization at MAMs would be very interesting, the key control of Miner reconstitution of Miner1-/- cells is missing. It is therefore impossible to causally link the observed phenotypes to lack of Miner1. In addition, the role of Mitoneet must be addressed biologically by ablating it in Miner1-/- cells and by performing and epistatic analysis of Miner1 and mitoneet. Without these important data, the paper falls short of providing convincing evidence for a role of Miner at the interface between mitochondria and ER and in all the downstream processes described here.

Specific points:

1. In fig.1A authors show a tissue fractionation experiment to obtain pure fractions of ER and mitochondria. They show purity controls for ER and mitochondria, but not for MAMs. Calnexin is not a MAMs markers because it is found also in ER. Authors need to show western blot against e.g. FACL4, which is known to be part of MAMs.
2. In fig.1B authors try to confirm ER localization by immunofluorescence in COS-7 cells. Again, Calnexin is not a suitable MAMs markers. Unfortunately the images are also overexposed and the signal is saturated: in such images it is not possible to distinguish ER from mitochondria. Authors needs to perform a triple immunofluorescence for ER, mitochondria and Miner1 as well as a specific colocalization between an established MAM marker and Miner1.

3. An additional confusing point needs to be clarified. Authors use two different constructs to define the Miner1 localization: EGFP-Miner1 and Miner1-EGFP. The first one localizes on mitochondria and the second one on ER. The explanation given by authors it is not sufficient to convince the reviewer and the reader that the right Miner1 localization is the ER. Authors need to show Miner1 localization by using a different tag. This is important also because previous reports described Miner1 to be located on mitochondria (Chen YF et al. 2009).
4. In fig. 3 authors try to demonstrate a dysregulation of calcium homeostasis by measuring calcium in ER and mitochondria. To this end, they use Miner1 WT and KO MEFs. Measurements of ER calcium with Fura-2 needs to be completed showing recover (total or partial) of calcium after Miner1 re-expression. When they switch to mitochondrial calcium measurement, the use of Calcium green 5N is troublesome because we cannot be sure that authors measure only mitochondrial calcium. In any case, to measure total mitochondrial calcium authors need to measure the total releasable calcium by treating mitochondria with alamethicin followed by calcium pulses to be sure that the probe is not saturated. These experiments must also be accompanied by ratiometric Ca2+ imaging in cytoplasm, ER and mitochondria using targeted chameleons or bioluminescent aequorin probes. Finally, the key experiment of Miner 1 re-expression in Miner1 -/- MEFs is missint.
5. In Fig.4 authors describe several Seahorse bioenergetic measurements comparing Miner1 WT and -/- MEFs. Several issues complicate the interpretation of these results:
5.1. Miner 1 -/- cells have a much higher oxygen consumption and respond also much more to FCCP. This could be due just to a higher number of cells than in control. To normalize the results I suggest that the authors count the cells in the plate after the measurements by staining them with a fluroescent probe (e.g. calcein) and taking several images at microscope. Same issue applies to Fig.6 C.
5.2. In addition, during the experiment it is necessary to wait a little bit longer between addition of oligo, FCCP or Rot/Ant A to see that the signal stabilize (see Seahorse webpage). I suggest that authors show 3 points (15 min) between additions to show the instrument stabilization.
5.3. Again, all the experiments of Miner1 reintroduction are missing
6. Fig.4.B authors measure oxygen consumption in isolated mitochondria from Miner1 WT and KO MEFs by Seahorse. They show the uncoupled respiration in presence of glutamate/Malate or succinate/rotenone as substrate. This experiment shall be replaced by a classical Clarke oxymetry with defined state 2, 3 4 and 4u measurements, as well as with P:O calculations to be meaningful.
7. Looking at the EM images from Miner1 KO MEFs it is not clear to me whether these cells have less mitochondria cristae. The image in supporting information Fig.3 E seems to present cristae that would be visible in the next section from the same sample. I would like to see serial section images. In any case, morphometry is required before concluding anything on the surface occupied by the cristae.
8. Fig.5 As a control for the specificity of the probe it is necessary to show that addition of NAC reduces the signal (Fig 5B-C).

Referee #3 (Comments on Novelty/Model System):

This is a very interesting paper about a system which is very novel and potentially of great interest. the work is extensive and in general well done.

Referee #3 (Other Remarks):

I found this a very interesting paper about a very intriguing and potentially very important protein. there is a great deal of work here and the quality of the work is high.

I do inevitably perhaps have some comments and a few concerns about some aspects of the work which I will describe simply in the order in which they appear in the manuscript.

1. the abstract is very important as this is what most people will read. While I appreciate that the word limit makes it hard, there are several rather vague comments that are not terribly useful and need to be clarified:

thus:
Abstract - 'miner deficient fibroblasts' - make it clear where these come from.

It isn't clear to me what a 'more oxidised milieu' means - might be better to be specific - oxidized GSH? Oxidised NADH suggests and oxidised mitochondrial milieu...?
'ultrastructral alterations' means what? I think this also needs to be specified.

2. NIDDM needs to be specified.
3. P4 'exquisitely sensitive to protonation at low pH', how low?

4. Calcium signals - the discrepancy between the responses to thapsigargin and to histamine is weird and needs investigation. The statement: 'possibly
indicating that the cells lacking Miner1 are primed for ER Ca2+ efflux' doesn't really make much sense and doesn't really address the issue. You simply can't argue that the ER Ca content is reduced when the histamine response is increased. These experiments need to be done in the absence of external calcium to distinguish capacitative influx from ER release. As oxidation increases Ca release it would be important to know what happens to these responses after treatment with NAC. the concentrations of thapsigargin and histamine need to be shown also in the figure legend at least.

The section on Mito Ca is not great:...., the statement 'and agents that induce the release of Ca2+ from the mitochondria' - this needs spelling out - what was actually used and what is its significance? The methods used here and the means to obtain the histogram shown are obscure and need to be explained properly.

'In fact, the total cellular Ca2+ content' - measured how? This remains poorly explained.

5. Is the increased OCR due to PDH activation? If so, DCA should stimulate both to the same absolute level and so make a smaller impact on the k/o.

6. You might also imagine an increase ATP consumption due to Ca cycling - in isolated mitochondria, is this RuR sensitive or modulated by a calcium chelator?

7. seeing it is an easy measurement to make, I am puzzled that there is no measurement of Mitochondrial potential ?

8. The increased NAD/NADH ratio is consistent with a higher OCR. It seems misleading to refer to this as 'intracellular milieu' as it is mostly a mitochondrial signal.

9. does NAC restore the ER Ca content?

Figures:
1. you MUST use a figure in which the signal is not saturating - you are missing data here.

2. NAC restores oxygen consumption characteristics a bit but this seems a bit exaggerated in the text.

Overall, the scheme given at the end is interesting but I'm not sure that the data really show conclusively 'what comes first'. If the final scheme is correct, you should improve the phenotype n several senses with Ca chelator.

1st Revision - authors' response 01 August 2012

Comments from Dr. Carret:

Thank you for the submission of your manuscript to EMBO Molecular Medicine. We have now heard back from the three referees whom we asked to evaluate your manuscript. Although the referees find the study to be of potential interest, they also raise a number of concerns that should be addressed in a major revision of the work.

As you will see, the referees find the study novel and interesting. However, they do suggest a certain number of additional experiments to strengthen the conclusions that I would encourage you to add.

Of particular relevance, a causal evidence for a direct role of Miner1 in the redox status of the cell should be provided as highlighted by Referee #1 and #2. These two reviewers also suggest performing a MitoNEET over-expression experiment to biologically address the role of MitoNEET. Referee #1 also suggests providing in vivo data to increase the physiological relevance. Finally a more thorough characterization of ER and mitochondria calcium homeostasis would improve the data.

As requested, we have performed the Miner1 rescue experiment (added as Fig. 7, which replaces the schematic of the proposed sequence of pathological changes in response to Miner1 deficiency (now included as Supporting Fig. 5). We discovered that re-expression of Miner1 does reverse multiple aspects of the KO phenotype. We have also experimentally addressed whether the Miner1 KO phenotype results from up-regulation of mitoNEET. We have tested the effects of both down-regulation and overexpression of mitoNEET in the Miner1 KO cells, and these results are presented in Supporting Information Figures 3 and 4. Importantly, mitoNEET overexpression did not recapitulate the phenotype of the Miner1 KO cells (Supporting Information Figure 4). We agree that providing in vivo data is the next step for this study, but believe that it is beyond the scope of the current, more mechanistic study.

Although we realize that all the requirements of Referee #2 might not all be necessary, I would appreciate if you could try to address as much as you can but in any case please reply to all reviewer's comments in writing.

We've done our best to address as many of Referee #2's concerns as we deemed reasonable and possible. All our responses are detailed below.

Referee #1:

In this study the authors show that Miner1-deficiency in MEF cells result in increased UPR markers, defects in mitochondrial respiration and increase in mitochondrial Ca++ load. The investigators also provide evidence supporting that Miner1-deficiency results in increased ROS production and creates an oxidized milieu as indicated by an increase in oxidized NAD+ and Gluthatione, therefore suggesting a role for direct influence of Miner1 in cellular redox status. The authors relate the increased Ca++ to an oxidative stress induced Ca++ leakage model, which they produce evidence to support this model with treatment of Miner1-deficient cells with NAC. This antioxidant treatment reduces CHOP and Bip levels and rescues the mitochondria phenotype in Miner1 deficient cells. Overall, the findings in this paper are extremely interesting. The results are novel and important and the paper is written well. Some aspects of the study would benefit from further work especially in generating a more thorough characterization of ER and mitochondria calcium homeostasis and a better morphological and/or cell biological analysis following antioxidant treatment. It would also be extremely useful to attempt to demonstrate MitoNEET up regulation or generate another line of more direct evidence to link Miner1-deficiency to the observed phenotype and exclude any potential contributions from MitoNEET overexpression in the experimental system used in the study. Otherwise, this is a high quality study with important information

Specific points for consideration:

1) The main evidence provided in supporting oxidative stress being upstream of calcium leak from ER is the NAC treatment experiments provided in Figure 6, in which no calcium biology was presented. I believe this work can be significantly strengthened if the authors can provide a more thorough characterization of ER and mitochondria calcium homeostasis and their morphologies

post-NAC treatment. It is important to establish that NAC treatment does reduce calcium leakage from ER and thus restore ER and calcium mitochondria homeostasis. It has also been previously shown that Ero1a can interact with IP3R to regulate calcium transport from ER to mitochondria. It may be productive to explore whether this well established pathway is involved in the observations presented here. In addition, the seahorse experiment with isolated mitochondria from Miner1-deficient cells (Figure 4D) exhibits higher OCR than those from WT cells, suggesting that the mitochondria function maybe independent of ER calcium release. Can the authors elaborate on the possible mechanism?

As part of the new Fig. 7, we show that either Miner1 re-expression or treatment with NAC normalizes the mitochondrial Ca^{2+} pool within the KO cells (Fig. 7D). We used a different approach (Rhod2) than previously employed to quantitate mitochondrial Ca^{2+} levels, which lends confidence that our previous method based on measurement of releasable Ca^{2+} from the matrix was providing reliable information. Although we attempted to monitor whether NAC also normalizes the ER Ca^{2+} pool, the treatment significantly altered the thapsigargin response in the wild type cells, making interpretation of the data difficult. This may have related to either an NAC-induced change in Ca^{2+} pools or in the behaviour of the Ca^{2+} sensitive dye.

With regard to the suggested line of experimentation into Ero1, we are appreciative of the suggestion to explore involvement of this clearly important protein. However, redox regulatory events involving Ero1/Pdi1p take place in the lumen of the ER, and the redox-active Fe-S cluster of Miner1 faces the cytoplasm, so it is not immediately obvious to us that a direct interplay of these proteins is likely. Regardless, this is an interesting line of investigation that we would like to pursue in the future.

The reviewer suggests that since the altered respiratory effects of the mitochondria persist after isolation, the elevated respiration must be independent of ER calcium release. We would respectfully disagree with this conclusion, as we believe that chronic changes in ER function could result in the persistent alterations in protein expression and cristae density (Fig. 4C and E) that we now appreciate can be reversed with Miner1 re-expression (Fig. 7).

2) The authors attribute all the phenotypes of Miner1-deficient cells to Miner1 depletion in the cell. As shown in Figure 1, 2 and 4, a major consequence of Miner1 depletion is MitoNEET up regulation. As MitoNEET is localized to mitochondria and MAM, could it be possible that the observed phenotypes may be influenced by this mechanism? The authors may consider performing a MitoNEET overexpression experiment to support or refute this possibility.

At the suggestion of Reviewer 2, we have performed a more rigorous MAM isolation and included FACL4 as a MAM marker protein (new Figure 1A). It is clear from this data that, contrary to our initial findings, mitoNEET is not localized to the MAMs but exclusively to the mitochondria. In contrast, Miner1 is clearly not in the mitochondria but in the microsomal ER and the MAMs. This makes it somewhat unlikely that the observed phenotype is due to the up regulation of mitoNEET. However, at the suggestion of the reviewers, we have ruled out contribution of mitoNEET up regulation by generating Miner1 WT and KO MEFs with a DOX inducible mitoNEET shRNA to lower mitoNEET levels. In addition, we have generated Miner1 WT MEFs that overexpress mitoNEET. We have analysed these cells for OCR, pPDH status, mitochondrial morphology, and ER stress and have found that the levels of mitoNEET do not appear to be contributing to the observed phenotype (Figure S3).

3) Rescue experiments with Miner1 re-introduction back into Miner1-/- cells would provide strong causal evidence for direct involvement of Miner1 in general redox status of the cell. Also a Miner1 mutant protein lacking (or mutated) CDGSH domain would further strengthen the argument for Miner1's role in regulating general redox status of the cell.

We have generated Miner1(-/-) MEFs with CMV-Miner1 reintroduced with a lentivirus and performed experiments to analyse parameters related to ER and mitochondrial homeostasis (Figure S4). The re-expression of Miner1 in these cells is far less than that of the Miner1(+/+) cells; however, the modest Miner1 expression in the Miner1(-/-) MEFs is sufficient to normalize the oxygen consumption and PDH phosphorylation status. In addition, ER stress markers are reduced (CHOP protein expression and tXBP1 gene expression). We did not re-express Miner1 lacking or with a mutated CDGSH domain in this study due to time constraints. Since Miner1 consists of the ER targeting sequence/TM domain and the CDGSH domain, omitting the CDGSH domain would leave only the targeting sequence, which we predict would be unstable. This would effectively mimic the frame shift mutations reported in human Wolfram Syndrome patients that result in a lack of any detectable protein.

4) In vivo studies involving Miner1-deficient models treated with NAC (or any other antioxidant) would strengthen the core findings of the study.

This would indeed be an exciting next step. Performing these experiments in our lab in the USA has been hindered by an inability to ship the Miner1(-/-) animals from Taiwan to the USA. These experiments may go forward in the lab of our collaborator, Dr. Tsai, who established the colony.

Minor points:

1) Figure 2D, the authors used calreticulin as a marker of ER and ER expansion. ER expansion was not noticeable on the EM level, just swelling. Is it possible that, under such severe ER stress condition (swollen ER), calreticulin is not exclusively restricted to the ER? Could this be tested?

There have been reports of calreticulin also localizing to the nucleus and binding to nuclear receptors; however, there is no obvious indication that the calreticulin in the Miner1 KO cells had any noticeable nuclear localization. The pattern was reticular with the individual tubules of the reticulum appear thicker than WT. We think that these data are consistent with ER expansion.

2) The authors showed that Bip level is increased as hallmark of ER stress, what happens to the level of calreticulin, XBP1 splicing or other UPR indicators?

Calreticulin protein is increased, as evidenced by the immunofluorescence staining (Figure 2D). XBP1 gene expression is increased (Figure 2B). We also reported elevated CHOP gene expression (Figure 2B) and protein levels (Figure 2C). We examined other ER stress indicators (PDI, Erdj, and Gadd34) by qRT-PCR but have not included this data because it was unremarkable.

3) Page 13, line#4 from bottom, "suggesting an enhanced ATP utilization", the data may also support enhanced ATP production instead. It is the combination of "enhanced ATP production" but "increased "ADP/ATP ratio" which would support ATP utilization is also enhanced.

We have reworded this sentence as the reviewer suggests.

4) Page 16, line 9, "Because the oxidative shift in the Miner1 KO cells was subtle...". Why do the authors thing these are subtle changes? Data presented in Figure 5B &C seem quite clear-cut to this reviewer. Can the data be quantified?

In an attempt to be conservative with our choice of adjectives, we used the term “subtle” only

because the thiol measurements show only a 20% change. We agree that the NO & ROS dye measurements were not subtle and were very clear-cut.

5) Can Authors also provide the absolute values of each nucleotide measured? Since the ratio of oxidized to/reduced nucleotides is critical and that oxidation is also a function of reduced pool availability; it would be important to see whether the reduced nucleotides are comparable in both cell lines. Could authors also check NADP+/NADPH levels, as there could be hydride transfers between these two species of dinucleotides.

The data we have for NAD^+/NADH ratios is relative to the concentration of each per volume of cell extract, not relative to protein concentrations. The sample is split & used for total & reduced (post NAD^+ decomposition) measurements; therefore, getting the ratio does not depend on the protein concentration of the samples. NAD^+ is calculated by subtracting NADH from total (NADt) NAD^+ + NADH. Starting with equal cell numbers and identical lysate volumes, in 50 µl of lysate, the WT had 270 pmol of NADt and 40 pmol of NADH, whereas the KO cells had 263 pmol of NADt and 32 pmol of NADH. The total pool of NAD^+ is lower in the KO, as well as the amount of reduced NADH. We attempted to measure $NADP^+$/NADPH ratios; however, the assay we used was not sensitive enough to measure the NADP levels in these MEF cells.

6) In multiple Figures, the author showed Bip is absent in WT cells. Bip is one of the most abundant proteins in the ER regardless of the status of ER and presence of stress.

Bip isn't actually absent in the WT cells. There is so much more Bip in KO cells that we used very short exposure times to show the Bip signal in the KO cells in the linear range. The WT signal wasn't visible with these exposure times.

7) As NAC treatment could have secondary/off target effects it would be highly valuable to try a different ROS scavenger to strengthen the argument in support of oxidative stress model? Alternatively a genetic model of antioxidant gene over expression in these cells (e.g. Glutathione reductase) could also be tried.

We initially tried the antioxidant Trolox, which did not reverse the ER stress. It is possible that the antioxidant needs to specifically affect the sulfhydryl redox status to be effective. We agree that it would be very interesting to try the glutathione reductase over-expression. Given the potential for complex effects of introducing another genetic variable to the system, we did not feel that we would be able to establish this system and characterize it under the time constraints of resubmission.

Referee #2:

In this paper authors investigate the subcellular localization of Miner1, and its role in cellular redox state. They claim that the enhanced oxidative intracellular environment of Miner1-/- cells induces general changes in the sulfhydryl redox state of the cell. As consequence, ER Ca^{2+} transporters show altered activity that results in decreased calcium storage in ER and increased calcium load in mitochondria. They further describe bioenergetic and morphological mitochondrial alteration in Miner1-/- MEFs, and accumulation of ROS. In general, even if Miner1 localization at MAMs would be very interesting, the key control of Miner reconstitution of Miner1-/- cells is missing. It is therefore impossible to causally link the observed phenotypes to lack of Miner1. In addition, the role of MitoNeet must be addressed biologically by ablating it in Miner1-/- cells and by performing and epistatic analysis of Miner1 and mitoNeet. Without these important data, the paper falls short of providing convincing evidence for a role of Miner at the interface between

mitochondria and ER and in all the downstream processes described here.

Specific points:

1. In fig.1A authors show a tissue fractionation experiment to obtain pure fractions of ER and mitochondria. They show purity controls for ER and mitochondria, but not for MAMs. Calnexin is not a MAMs markers because it is found also in ER. Authors need to show western blot against e.g. FACL4, which is known to be part of MAMs.

We are indebted to Reviewer 2 for requesting a more thorough assessment of the MAM fractions. We actually utilized a different isolation protocol that is more rigorous and gives very clean MAM fractions. In addition, we have used FACL4 as a MAM marker in the Western blots, as the reviewer suggested. This data is present in the new Fig 1A. Our initial fractionation suggested that mitoNEET was also present in the MAM fraction; however, FACL4 is highly enriched in the MAM fraction (along with Miner1), but there is no mitoNEET present in this fraction. Therefore, this protocol change has allowed us to streamline the explanation of this figure and make the data more clear-cut.

2. In fig.1B authors try to confirm ER localization by immunofluorescence in COS-7 cells. Again, Calnexin is not a suitable MAMs markers. Unfortunately the images are also overexposed and the signal is saturated: in such images it is not possible to distinguish ER from mitochondria. Authors needs to perform a triple immunofluorescence for ER, mitochondria and Miner1 as well as a specific colocalization between an established MAM marker and Miner1.

Calnexin was not used as a MAM marker here, rather it was chosen as just a general marker for ER. The signal is overexposed to allow visualization of the reticulum & not just the perinuclear region, since Miner1 is not exclusively at the MAM. We have previously published costaining with Miner1-EGFP and MitoTracker Red and demonstrated that they do not colocalize. We attempted to do co-localization with Miner1-GFP and FACL4 (MAM marker); however, we could not find an antibody that worked for immunostaining. This figure was included to strengthen the ER localization claim, since others have published immunofluorescence localization studies suggesting mitochondrial localization for Miner1. We felt it was important to experimentally address this debated issue.

3. An additional confusing point needs to be clarified. Authors use two different constructs to define the Miner1 localization: EGFP-Miner1 and Miner1-EGFP. The first one localizes on mitochondria and the second one on ER. The explanation given by authors it is not sufficient to convince the reviewer and the reader that the right Miner1 localization is the ER. Authors need to show Miner1 localization by using a different tag. This is important also because previous reports described Miner1 to be located on mitochondria (Chen YF et al. 2009).

We initially included this data because we felt it was important to clarify the discrepancy between our immunofluorescence localization and the report by Chen that the reviewer mentions, which describes a mitochondrial localization (or primarily mitochondrial with a minor portion at the ER) for Miner1. It seems that we only confused the issue, so we have removed the supplemental figure with this immunofluorescence data.

We have previously reported Miner1 immunolocalization using a small V-5 carboxy-terminal epitope tag. The pattern was that of the ER and did not colocalize with MitoTracker Red. The Miner1-EGFP also localizes to the ER and not the mitochondria.

We feel that the current Fig 1A with the vastly improved MAM fraction and highly purified

mitochondrial fraction settles this issue of Miner1 ER localization. The paper by Chen (2009) suggests that Miner1 is localized to the mitochondria based on a crude mitochondrial fraction (where we also see ample Miner1 signal, Fig 1A). The mitochondria were not gradient-purified in that study and there were no ER markers presented to demonstrate purity of the fractions. Both Chen and Tsai (the senior author of the 2009 manuscript describing the Miner1 knockout mice) are co-authors on this manuscript and do not object to our conclusions.

4. In fig. 3 authors try to demonstrate a dysregulation of calcium homeostasis by measuring calcium in ER and mitochondria. To this end, they use Miner1 WT and KO MEFs. Measurements of ER calcium with Fura-2 need to be completed showing recover (total or partial) of calcium after Miner1 re-expression. When they switch to mitochondrial calcium measurement, the use of Calcium green 5N is troublesome because we cannot be sure that authors measure only mitochondrial calcium. In any case, to measure total mitochondrial calcium authors need to measure the total releasable calcium by treating mitochondria with alamethicin followed by calcium pulses to be sure that the probe is not saturated. These experiments must also be accompanied by ratiometric Ca2+ imaging in cytoplasm, ER and mitochondria using targeted chameleons or bioluminescent aequorin probes. Finally, the key experiment of Miner 1 re-expression in Miner1 -/- MEFs is missing.

We agree that it is critical to ensure that the Calcium Green probe is not saturated. The experimental design was explained in detail in the Supplemental Information section but somewhat glossed over in the main body of the text. We show a representative trace of the raw data in Supporting Information Figure 1, demonstrating the addition of Alamethicin as well as pulses of Ca^{2+} to each run. In addition, this is explained in detail in the Supplemental Methods section. We have added a few sentences to the main text highlighting the addition of Alamethicin and Ca^{2+} in this experiment and hope that this clarifies the issue.

5. In Fig.4 authors describe several Seahorse bioenergetic measurements comparing Miner1 WT and -/- MEFs. Several issues complicate the interpretation of these results:

5.1. Miner 1 -/- cells have a much higher oxygen consumption and respond also much more to FCCP. This could be due just to a higher number of cells than in control. To normalize the results I suggest that the authors count the cells in the plate after the measurements by staining them with a fluorescent probe (e.g. calcein) and taking several images at microscope. Same issue applies to Fig.6 C.

Cells are counted minutes prior to each run in the Seahorse and spun onto the plate with Cell Tak as described in the Supplemental Methods. Therefore, we do not believe that differences in cell number are responsible for the rates observed. Unfortunately, there are no fluorescent methodologies at present that give linear reliable quantitation of the cells on the Seahorse plate post-run. Our lab works closely with the Seahorse Biosciences to develop and test new tools for use in the Seahorse and recognize this as an important issue for cells that have been maintained in the plate for extended periods of time, unlike the approach employed for our data.

5.2. In addition, during the experiment it is necessary to wait a little bit longer between addition of oligo, FCCP or Rot/Ant A to see that the signal stabilize (see Seahorse webpage). I suggest that authors show 3 points (15 min) between additions to show the instrument stabilization.

Our lab at UCSD hosts 2 visiting research & development scientists from Seahorse; consequently, our 3 Seahorse machines are regularly serviced and assessed for stability of signal and performance. The parameters that we used for running these experiments were determined to give reliable & reproducible results without resulting in loss of the cells from the Cell-Tak substratum. The period over which a single data point is generated actually represents multiple measurements (once every 16 seconds) over at least a 2-minute period or more, which is similar in duration to a reading in a conventional electrode chamber.

5.3. Again, all the experiments of Miner1 reintroduction are missing

We have added Figure 7 demonstrating that reintroduction of even a modest amount of Miner1 into the Miner1 -/- MEFs results in restoring OCR to levels of those of Miner1 WT cells.

6. Fig.4.B authors measure oxygen consumption in isolated mitochondria from Miner1 WT and KO MEFs by Seahorse. They show the uncoupled respiration in presence of glutamate/Malate or succinate/rotenone as substrate. This experiment shall be replaced by a classical Clarke oxymetry with defined state 2, 3 4 and 4u measurements, as well as with P:O calculations to be meaningful.

We describe the protocol for measuring OCR in isolated mitochondria using Seahorse technology in the Supporting Methods. We have recently published the development of this methodology in a peer-reviewed journal (Rogers et al, 2010) and routinely sequentially measure state 3, state 4, and uncoupler-stimulated rates. Given the ability to run replicate samples with multiple substrates simultaneously, this method has many advantages over classical Clarke oxymetry while maintaining sensitivity. Since the greatest differences between Miner1 WT and -/- whole cell OCRs was in the uncoupler-stimulated (FCCP) rate measurements, we think that the most relevant data for comparison is the FCCP-stimulated maximal rates for the isolated mitochondria. We believe that the data are meaningful as shown. In general, we choose not report P:O ratios, as the method of measurement is inherently flawed. The proton leak is non-Ohmic, thus the rate of leak in State 3 and State 4 is not equivalent, which skews the estimate of the number of ATP produced per oxygen consumed.

7. Looking at the EM images from Miner1 KO MEFs it is not clear to me whether these cells have less mitochondria cristae. The image in supporting information Fig.3 E seems to present cristae that would be visible in the next section from the same sample. I would like to see serial section images. In any case, morphometry is required before concluding anything on the surface occupied by the cristae.

We agree with the reviewer that morphometry using EM is needed to make conclusions regarding cristae abundance. We now include these morphometric data in Fig 4E, demonstrating an *increase* in cristae abundance in the Miner1 KO MEFs, as well as in Supporting information Fig 2D, which shows that there is no difference in the volume of mitochondria per cell in Miner1 WT and KO MEFs. We have changed the methods section describing these experiments to clarify that morphometric analysis was performed on the EM images.

8. Fig.5 As a control for the specificity of the probe it is necessary to show that addition of NAC reduces the signal (Fig 5B-C).

We believe that specificity of the probe has been previously established.

Referee #3 (Comments on Novelty/Model System):

This is a very interesting paper about a system, which is very novel, and potentially of great interest. the work is extensive and in general well done.

Referee #3 (Other Remarks):

I found this a very interesting paper about a very intriguing and potentially very important protein. There is a great deal of work here and the quality of the work is high.

I do inevitably perhaps have some comments and a few concerns about some aspects of the work, which I will describe simply in the order in which they appear in the manuscript.

1. the abstract is very important as this is what most people will read. While I appreciate that the word limit makes it hard, there are several rather vague comments that are not terribly useful and need to be clarified:

thus:

Abstract - 'miner deficient fibroblasts' - make it clear where these come from.

It isn't clear to me what a 'more oxidised milieu' means - might be better to be specific - oxidized GSH? Oxidised NADH suggests and oxidised mitochondrial milieu...?

'ultrastructral alterations' means what? I think this also needs to be specified.

We have altered the abstract to incorporate the reviewer's suggestions to make the abstract more specific.

2. NIDDM needs to be specified.

We have defined NIDDM in the text.

3. P4 'exquisitely sensitive to protonation at low pH', how low?

The 2Fe2S cluster ligated by the CDGSH domain begins to be released from the CDGSH even at pH 7.0, however the rate increases dramatically as the pH is lowered below neutral. In contrast, we reported that ferredoxin binds its 2Fe2S cluster even at pH4. We have clarified this in the text.

4. Calcium signals - the discrepancy between the responses to thapsigargin and to histamine is weird and needs investigation. The statement: 'possibly indicating that the cells lacking Miner1 are primed for ER Ca2+ efflux' doesn't really make much sense and doesn't really address the issue. You simply can't argue that the ER Ca content is reduced when the histamine response is increased. These experiments need to be done in the absence of external calcium to distinguish capacitative influx from ER release. As oxidation increases Ca release it would be important to know what happens to these responses after treatment with NAC. The concentrations of thapsigargin and histamine need to be shown also in the figure legend at least.

The section on Mito Ca is not great: the statement 'and agents that induce the release of Ca2+ from the mitochondria' - this needs spelling out - what was actually used and what is its significance? The methods used here and the means to obtain the histogram shown are obscure and need to be explained properly.

'In fact, the total cellular Ca2+ content' - measured how? This remains poorly explained.

We have attempted to clarify the methodology in the main text, in addition to the descriptions of the

experiments that was in the Supporting information. The thapsigargin-induced Ca^{2+} release yielded similar results with EGTA included to chelate external Ca^{2+}.

5. Is the increased OCR due to PDH activation? If so, DCA should stimulate both to the same absolute level and so make a smaller impact on the k/o.

We presume that a portion of the increased OCR may be due to PDH activation. However, there are other TCA cycle dehydrogenases that are also stimulated by Ca^{2+}. It is unlikely that the enhanced OCR is the function of a change in only one enzyme activity, as there is an increase in cristae density and ETC proteins (Fig. 4), possibly indicating increased functional ETC protein complexes.

6. You might also imagine an increase ATP consumption due to Ca cycling - in isolated mitochondria, is this RuR sensitive or modulated by a calcium chelator?

We agree that the increased ATP consumption could very likely be due to Ca^{2+} cycling. We have not tested whether it is RuR sensitive in isolated mitochondria, since we envision the Ca2+ cycling to be between the ER and the mitochondria, with the ER Ca2+ ATPases being major consumers of ATP. Increased mitochondrial Ca^{2+} cycling would be reflected in an increase in state 4 respiration, which is not dramatically altered in the cells (Fig. 4).

7. Seeing it is an easy measurement to make, I am puzzled that there is no measurement of Mitochondrial potential ?

Making comparative measurements of membrane potential is possible, although somewhat difficult when cell morphology (and thus dye loading, and nonspecific binding) has changed. We believe that the combination of oxygen consumption, ADP/ATP, and NAD^+/NADH ratios we report gives a fairly robust picture of the cellular bioenergetics.

8. The increased NAD/NADH ratio is consistent with a higher OCR. It seems misleading to refer to this as 'intracellular milieu' as it is mostly a mitochondrial signal.

We were using ‘intracellular milieu’ to include ROS and RNS, reduced thiols, etc, in essence all of the data in this section combined, which interrogates signals from the mitochondria as well as the cytosol, but for clarity, this wording in the abstract has been replaced with more specific descriptors.

9. Does NAC restore the ER Ca content?

Although we attempted this experiment, we found the results difficult to interpret, as NAC treatment significantly lowered the Ca^{2+} response in the WT cells. At this time, we do not know whether this relates to alteration of the ER pool, or alteration of behaviour of the Ca^{2+}-sensitive fluorophore.

Figures:

1. You MUST use a figure in which the signal is not saturating - you are missing data here.

Our goal with Fig 1B was to demonstrate the localization to the reticular structures in the cell, which unfortunately results in the perinuclear region being over saturated. We have previously published that Miner1-V5 does not colocalize with MitoTracker Red.

2. NAC restores oxygen consumption characteristics a bit but this seems a bit exaggerated in the text.

Overall, the scheme given at the end is interesting but I'm not sure that the data really show conclusively 'what comes first'. If the final scheme is correct, you should improve the phenotype n several senses with Ca chelator.

We are uncertain that acute treatment with a Ca^{2+} chelator would reverse longer-term responses of stress signalling, cristae and ETC complex levels, etc.

2nd Editorial Decision 06 September 2012

Thank you for the submission of your revised manuscript "Wolfram Syndrome protein, Miner1, regulates sulfhydryl redox status, the unfolded protein response and Ca2+ homeostasis" to EMBO Molecular Medicine.

We have now received the reports from the reviewers who were asked to re-review your manuscript. As you will see, the Reviewers acknowledge that the manuscript was significantly improved during revision and Reviewer #3 indicates that it is suitable for publication. However, Reviewer #2 still raises significant concerns about the conclusiveness of results. Since we do acknowledge the potential interest of your findings, we would be willing to consider a revised manuscript with the understanding that the referee concerns must be convincingly and conclusively addressed.

Importantly, Reviewer #2 points out that the assessment of calcium homeostasis should be improved. In addition, we would strongly encourage you to replace the current Fig 1B with a better quality figure that is not overexposed. Regarding the reviewers point 2, 4 and 5, we believe that these could be addressed in writing.

On a more editorial note, please address the concerns below:

- The description of all reported data that includes statistical testing must state the name of the statistical test used to generate error bars and P values, the number (n) of independent experiments underlying each data point (not replicate measures of one sample), and the actual P value for each test (not merely 'significant' or 'P < 0.05').

- Please include scale bars in the immunofluorescence and EM panels.

***** Reviewer's comments *****

Referee #2:

Authors performed some controls deemed essential in my previous review. However, I still have doubts on the following points, raised in my previous review
1. Fig.1B was not changed. Again, a triple immunofluorescence for ER, mitochondria and Miner1 is missing.
2. In Fig.7 the release of calcium in response to thapsigargin seems to be higher than the one shown in Fig. 3 (wt, 1700 versus 750nM, forKO 1200 versus 300nm). How do the authors explain this difference?
3. Ca2+ experiments. These are key experiments for the conclusions drawn by the Authors, so they shall be very much controlled and performed accurately. Authors measure mitochondrial calcium by using dihydro-Rhod 2-AM, which exhibits Ca2+-dependent fluorescence only after it is oxidized and this occurs preferentially in mitochondria. Thus, specificity of Rhod2 relies on mitochondrial potential, which was never measured in the manuscript . Moreover, authors show that Miner1-/-

have a oxidized intracellular milieu; finally, Rhod2 has a very low Kd and is therefore easily saturated at low intramitochondrial Ca2+ concentrations. For these reasons, dihydro-Rhod 2-AM is not the most suitable probe to use to measure mitochondrial calcium. Moreover for the experiment shown in figure 7, it is not clear how data are expressed and which stimulus was used to assess mitochondrial calcium content. Finally, the experiment should be accompanied by Ca2+ imaging in cytoplasm, as asked before. Again chameleons or aequorin probes should be used to assess calcium homeostasis in situ.
4. In their response, the Authors reinstate that they are knowledgeable in the bioenergetics measurements. I am convinced that they are, but I still feel that the experiments I proposed are essential. I disagree that the main difference observed in situ is in the FCCP stimulated respiration, since also the basal respiration differs between wt and Miner1-/- cells, for example.
5. morphometry of the cristae is still missing.

Referee #3:

Suitable for publication.

2nd Revision - authors' response 04 February 2013

Referee #1 & 3:

Reviewers 1 and 3 each raised 11 points in the initial review. We addressed all of their concerns in the first revision, and both of these reviewers found the revised version suitable for publication.

Referee #2:

Reviewer 2 raised 10 points in the initial review, which we made every attempt to address in the initial revision. However, reviewer 2 still expressed concerns with the manuscript. These are outlined below with our responses.

Authors performed some controls deemed essential in my previous review. However, I still have doubts on the following points, raised in my previous review.

1. Fig.1B was not changed. Again, a triple immunofluorescence for ER, mitochondria and Miner1 is missing.

We have replaced Fig. 1B with a new set of panels depicting co-staining of Miner1-EGFP and calreticulin to highlight the ER localization of Miner1. The individual images are shown in black and white for maximal detail of the reticular ER. The merged image is shown in color. In addition, we have added Supporting information Fig 1, which includes a triple stain of Miner1-EGFP, calreticulin, and cytochrome c. Supporting information Fig 2 shows the EGFP control images. We feel that these new images support our previously published data (Miner1-V5 and MitoTracker Red images that showed non-mitochondrial localization of Miner1). Together with the cellular subfractionation data presented in Fig. 1A, this clearly establishes the localization of Miner1 at the ER and MAM, as opposed to the mitochondria.

2. In Fig.7 the release of calcium in response to thapsigargin seems to be higher than the one shown in Fig. 3 (wt, 1700 versus 750nM, for KO 1200 versus 300nm). How do the authors explain this difference?

We believe the discrepancy in the responses was because the experiments in Fig. 7 were performed with double the cell numbers and a newer lot of the Ca2+ fluorophore. The cells used for these experiments had also been transduced with Luciferase control or Miner1 expressing lentivirus and selected as stable populations. It is possible that this could have altered the cell responsiveness; however, the relative differences between WT and KO are identical to the initial findings.

3. Ca^{2+} experiments. These are key experiments for the conclusions drawn by the Authors, so they shall be very much controlled and performed accurately. Authors measure mitochondrial calcium by using dihydro-Rhod 2-AM, which exhibits Ca^{2+}dependent fluorescence only after it is oxidized and this occurs preferentially in mitochondria. Thus, specificity of Rhod2 relies on mitochondrial potential, which was never measured in the manuscript. Moreover, authors show that Miner1-/- have a oxidized intracellular milieu; finally, Rhod2 has a very low Kd and is therefore easily saturated at low intramitochondrial Ca^{2+}concentrations. For these reasons, dihydro-Rhod 2-AM is not the most suitable probe to use to measure mitochondrial calcium. Moreover for the experiment shown in figure 7, it is not clear how data are expressed and which stimulus was used to assess mitochondrial calcium content. Finally, the experiment should be accompanied by Ca^{2+}imaging in cytoplasm, as asked before.

Again chameleons or aequorin probes should be used to assess calcium homeostasis in situ.

Reviewer 2 raises a valid point regarding the issues concerning dihydro-Rhod-2-AM to measure mitochondrial Ca2+. We used this approach as a second methodology to assess the mitochondrial Ca2+ content, in addition to the Calcium Green 5N method initially used for the WT and Miner1 KO cells that we report in Fig 3D. When we submitted the first revised manuscript, we had time constraints to complete the number of experiments requested by all reviewers, which required generating several lentiviral constructs and creating a variety of stable cell lines. We were unable to generate the numbers of cells necessary to perform the mito Ca2+ on the Miner1 rescue cells using our original method prior to resubmission, and chose the Rhod2 method as an alternative approach. We have now removed this figure panel and replaced it with a panel produced using the original methodology from Fig 3, and place the Rhod2 data into the supplement.

It must be stressed that our goal was to measure the total mitochondrial Ca2+ load, not the matrix free Ca2+ level. There was no trigger used in the Rhod2 experiments. We were not interested in monitoring the temporal dynamics of mitochondrial Ca2+ flux, which can be influenced by many factors.

Regardless, we attempted to use mitochondrially targeted aequorin (AEQ) to assess the basal mitochondrial free Ca2+, and meet the reviewer's request. Unfortunately, the company that sold targeted AEQ vectors does not seem to still be in operation, so we were not able to purchase constructs. We generously received a lentiviral mito-AEQ construct from Dr. Vamsi Mootha's lab, which was one of two labs that recently identified the mitochondrial Ca2+ uniporter.. After packaging virus and generating stable mito-AEQ expressing cell lines, we confirmed expression and responsiveness of the AEQ using a Tecan plate reader in luminescence mode, following a protocol provided by the Mootha Lab. All cell lines responded robustly to triggers such as ionomycin or histamine to induce a Ca2+ influx into the mitochondria; however, the response is exceedingly fast, as the reviewer is undoubtedly aware. We monitored the loading of the AEQ and could detect an initial very small increase in luminescence while loading; however it was rapidly extinguished. Targeted AEQ is a powerful tool for measuring trigger-induced temporal changes in matrix free Ca2+. However, this was not our objective.

Thus, this technique proved inadequate for our purposes, which was to monitor basal total mitochondrial Ca2+, or how much Ca2+ is "constitutively" loaded into the mitochondria due to the proposed on-going ER Ca2+ leak. Our desire to measure total Ca2+ is based on the fact that the majority of matrix Ca2+ exists as precipitated calcium and not as free matrix Ca2+; this is particularly true under conditions of extreme mitochondrial Ca2+ loading (Chalmers and Nicholls, J Biol Chem, 2003, May 23;278 and many others). These precipitates require a considerable length of

time (upwards of 20 minutes) to go back into solution once the mitochondria are induced to release Ca2+, such as with FCCP/Antimycin A as in our Calcium Green experiments. The ability to collect data over a long time course as the total pool of mitochondrial Ca2+ is released makes the Calcium Green method preferable to AEQ for this purpose.

4. In their response, the Authors reinstate that they are knowledgeable in the bioenergetics measurements. I am convinced that they are, but I still feel that the experiments I proposed are essential. I disagree that the main difference observed in situ is in the FCCP stimulated respiration, since also the basal respiration differs between wt and Miner1-/- cells, for example.

We must point out in our manuscript we made the specific point that there are increases in both basal and maximal FCCP-stimulated in the Miner1 -/- cells. The difference in the endogenous rate is quite important to the conclusions. It is what led us to the conclusion that ATP turnover (energy utilization) in the knockout cells is enhanced.

We shared the reviewer's earlier concerns regarding cell number accuracy, in our measurements, as large differences in cell number could potentially account for the differences in respiratory rates. To address this, we purposely designed our experiments to correct for any differences in growth rate.. Perhaps this detail of our experimental design escaped the reviewer's notice because it was described primarily in the Supporting Methods. We harvested cells immediately prior to each experiment, counted the cells, and attached the same number of cells to each Seahorse well using CellTak, obviating any issue with differences in cell number.

Regarding the mitochondrial respiration data presented in Fig. 4D, the reviewer initially requested that we repeat these experiments using "classic Clarke electrode oxymetry with defined state 2, 3, 4, and 4u measurements." The protocol for utilizing the Seahorse XF24 and XF96 to measure oxygen consumption in isolated mitochondria was developed by Dr. Murphy and Dr. Martin Brand in collaboration with Seahorse scientists, and was published in a peer-reviewed journal in 2011. In the review process, the approach was fully vetted by 3 bioenergeticists. It is already being widely utilized in the field. We have added the reference to the main body of the text. This technique is extremely valuable because it allows many replicates to be run simultaneously, as well as concurrent comparison of control and knockdown cells. With conventional oxymetry, there is typically too little material to perform multiple replicates, and the quality of mitochondria isolated from cultured cells declines with time after isolation, introducing error associated with the slow pace of conventional electrode measurement. We chose to focus on the FCCP-induced maximal rates for the isolated mitochondria experiment simply because the relative difference between WT and Miner1 -/- whole cell OCR were greatest with FCCP. Basal, state 4, and maximal uncoupled OCR for whole cells are depicted in Fig 4B. The intention of Fig 4D was not to re-establish differences in all of these OCR in isolated mitochondria, rather to demonstrate that the increases in OCR observed in the Miner1 -/- cells are also present at the level of the mitochondria, not just the whole cell level. Together, the data presented in Fig 4 B, C, D, E and Supporting information Fig 4 D collectively support the conclusion that the increased OCR in the Miner1 -/- cells is not due to an increase in mitochondria per cell but due to increased oxygen consumption per mg of mitochondria, possibly due to the increased cristae volume and increased expression of electron transport chain proteins.

5. morphometry of the cristae is still missing.

The cristae morphometry has been present as Fig 4E in every version of this manuscript. The methods and Figure Legend for Fig 4E in the main text describe the morphometry for the cristae quantitatively, based on images such as those shown in Supporting Info Fig 4E. We hope this clears up the confusion over this figure.

3rd Editorial Decision 08 March 2013

Thank you for the submission of your revised manuscript to EMBO Molecular Medicine. We have now received the enclosed report from the referee who was asked to re-assess it. This reviewer is now globally supportive and I am pleased to inform you that we will be able to accept your manuscript pending the following final amendments:

-Please reply to the referee's concerns as suggested
-Please address the following editorial matters:
1/ Figures: Please increase the labeling on histograms and add scale bars to the immunofluorescence images
2/ figure legends: please indicate when appropriate which statistical test was performed, n and p such as (statistical test, n=x, p=y)
3/ We now encourage the publication of source data, particularly for electrophoretic gels and blots, with the aim of making primary data more accessible and transparent to the reader. Would you be willing to provide a PDF file per figure that contains the original, uncropped and unprocessed scans of all or key gels used in the figure? The PDF files should be labeled with the appropriate figure/panel number, and should have molecular weight markers; further annotation may be useful but is not essential. The PDF files will be published online with the article as supplementary "Source Data" files. If you have any questions regarding this just contact me

***** Reviewer's comments *****

Referee #2:

I thank the authors for having addressed my main concerns. The following concerns remain and the authors shall address them in writing or just by revising their original data
1. Point 2. I don't agree. Ca2+ measurements using ratiometric Ca2+ probes are independent from the batch, the concentration or the loading of the dye. In addition, the relative difference is not the same as the authors say. I suggest that they go back to the original raw data and check the calibration
2. Point 3. I disagree with the explanation of the authors. GFP based ratiometric probes or aequorin are calibratable and therefore suited to measure basal free Ca2+ levels (much more important than total Ca2+: as the authors know very well the Ca2+-Pi precipitates are important only under extreme Ca2+ loads, their relative contribution to mitochondrial Ca2+ pools are heavily debated, and are in equilibrium with the free Ca2+ levels in the matrix). I suggest that the authors remove the Rhod2 data completely and discuss the potential issues of their measurements.

3rd Revision - authors' response 26 March 2013

Editor's comments:

Please reply to the referee's concerns as suggested

-Please address the following editorial matters:

1/ Figures: Please increase the labelling on histograms and add scale bars to the immunofluorescence images

There are now scale bars in the images in Figures 1B, Fig 2D, Supporting Information Figs 1, 2, and 4, and the level of magnification on images in Fig 5B and C have now been stated. We have attempted to clarify the definition of the labels in each of the figure legends.

2/ figure legends: please indicate when appropriate which statistical test was performed, n and p such as (statistical test, n=x, p=y)

© EMBO

The type of test applied, along with the n and p values are now given in the figure legends.

3/ We now encourage the publication of source data, particularly for electrophoretic gels and blots, with the aim of making primary data more accessible and transparent to the reader. Would you be willing to provide a PDF file per figure that contains the original, uncropped and unprocessed scans of all or key gels used in the figure? The PDF files should be labelled with the appropriate figure/panel number, and should have molecular weight markers; further annotation may be useful but is not essential. The PDF files will be published online with the article as supplementary "Source Data" files. If you have any questions regarding this just contact me.

We have provided some source data for the western blots, but I must say they are very 'raw' in appearance! Would you prefer that we make these more polished with replacement of hand written labels?

***** Reviewer's comments *****

Referee #2:

I thank the authors for having addressed my main concerns. The following concerns remain and the authors shall address them in writing or just by revising their original data

1. Point 2. I don't agree. Ca^{2+} measurements using ratiometric Ca^{2+} probes are independent from the batch, the concentration or the loading of the dye. In addition, the relative difference is not the same as the authors say. I suggest that they go back to the original raw data and check the calibration.

The reviewer is certainly correct that the ratiometric dye is not the source of the variable responsiveness in the two sets of experiments in question. As suggested, we have gone back to the original data and checked the calibration signals (minimum and maximal fluorescence values). The ratio of Fmax/Fmin was indeed constant (although the raw fluorescence values were higher for the experiments of Fig. 7 due to the higher cell number), so it is unlikely that there was a problem with the calibration. Again, we plated double the number of cells for the experiments in the rescue experiments in Fig. 7, and the cells were infected with lentivirus. Either one of these changes could contribute to alteration of the cell responsiveness to thapsigargin. The important and consistent point is that knockout of Miner1 has decreased the thapsigargin-releasable pool of Ca^{2+}, and re-expression of the protein significantly repletes this pool.

2. Point 3. I disagree with the explanation of the authors. GFP based ratiometric probes or aequorin are calibratable and therefore suited to measure basal free Ca^{2+} levels (much more important than total Ca^{2+}: as the authors know very well the Ca^{2+}-Pi precipitates are important only under extreme Ca^{2+} loads, their relative contribution to mitochondrial Ca^{2+} pools are heavily debated, and are in equilibrium with the free Ca^{2+} levels in the matrix). I suggest that the authors remove the Rhod2 data completely and discuss the potential issues of their measurements.

There is no doubt that basal free mitochondrial Ca^{2+} is more important than the precipitated pool under non-pathological conditions. As the reviewer states, the size of the precipitated pool is important under extreme Ca^{2+} loads that occur in pathological states. This, in fact, is the condition we believe that we are studying. The point of our manuscript is that indeed, the knockout of Miner1 creates a defect in cellular Ca^{2+} handling that likely does help explain the severe degenerative phenotype of the Miner1 knockout mouse from which the cells were derived. We believe this also sheds light on the severe degenerative condition in Wolfram Syndrome patients. We fully appreciate that free Ca^{2+} can be measured with calibratable probes, and admire those who do this well. We

simply disagree that this is the most telling measure in this model of cellular pathophysiology. A chronic leak of Ca^{2+} from the ER would be anticipated to result in extreme mitochondrial loading, and this load is what we have documented in our data, along with the observation that it is reversed with re-expression of Miner1. As addressed in the discussion, the fact that the knockout cells survive this dysfunction in culture is likely related to an adaptive response associated with the SV40 large T antigen-mediated transformation process of the embryonic fibroblasts. We have previously described that changes associated with cell transformation include an increase in the mitochondrial capacity to accumulate and retain large quantities of Ca^{2+} [Murphy et al, 1996, Bcl-2 potentiates the maximal calcium uptake capacity of neural cell mitochondria Proc. Natl. Acad. Sci. USA 93: 9893-9898; Murphy AN and Fiskum G. (1988) Abnormal calcium transport characteristics of hepatoma mitochondria and endoplasmic reticulum, In "Cellular Calcium Regulation" (Pfeiffer, D., McMillin, J. and Little, S., Eds.) Plenum Press Inc., New York, pp.139-150]. Overall, we stand by our contention that total mitochondrial Ca^{2+} is a relevant measure of the mechanistic changes induced by Miner1 deficiency. As suggested by the reviewer, we have removed the Rhod2 data from the manuscript.
