# Supplementary figures and images for: Wolfram Syndrome protein, Miner1, regulates sulphydryl redox status, the unfolded protein response, and Ca2+ homeostasis
Source: EMBO Mol Med. 2013 May 24;5(6):904–18. doi: 10.1002/emmm.201201429 (PMC3779451; doi:10.1002/emmm.201201429)

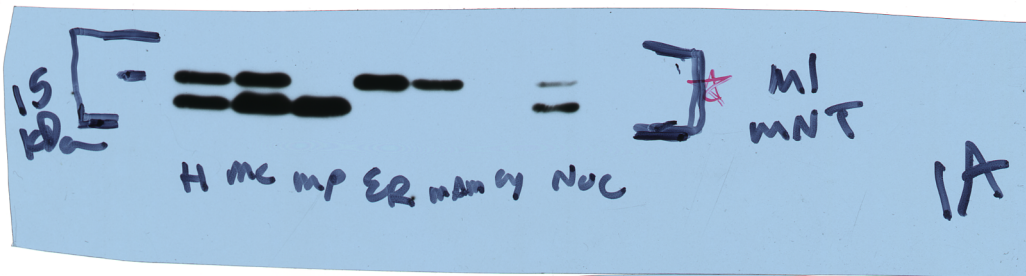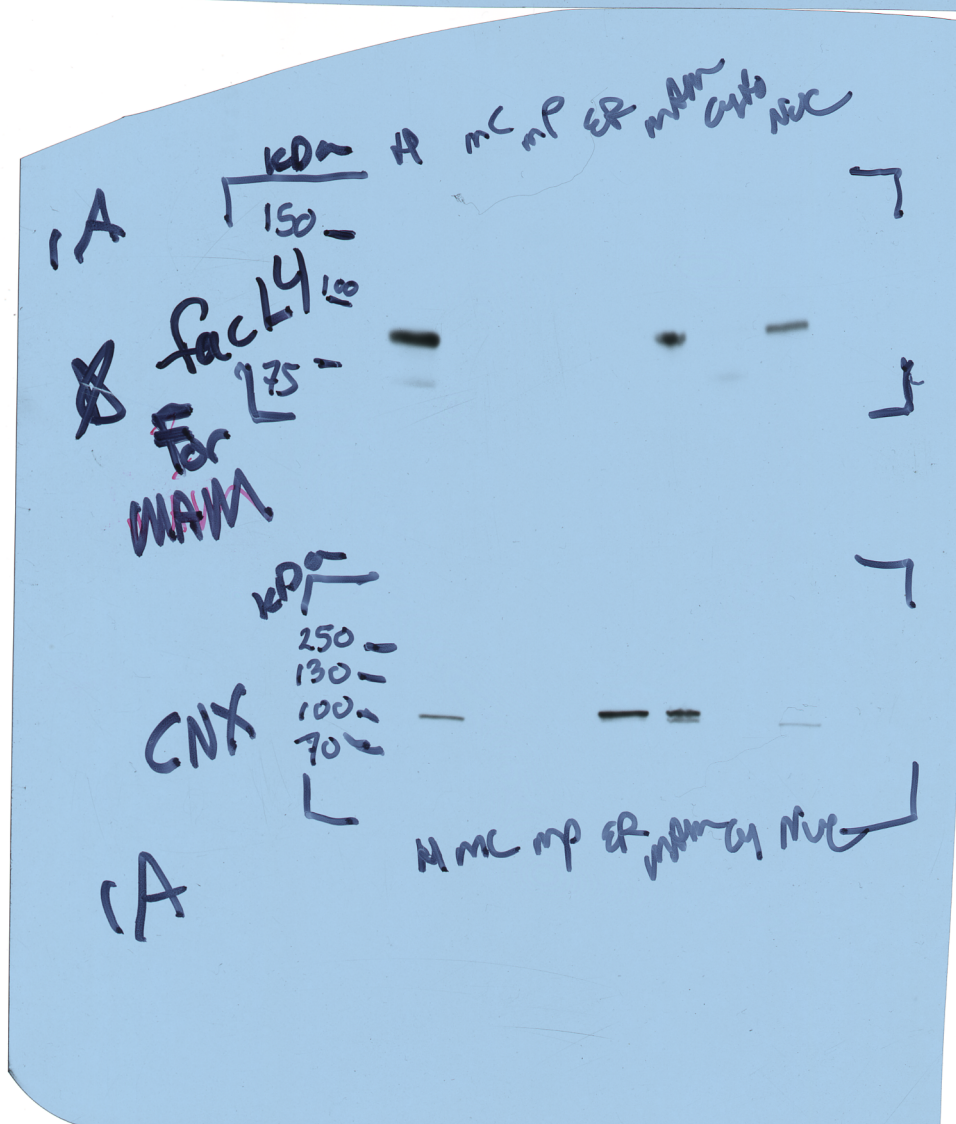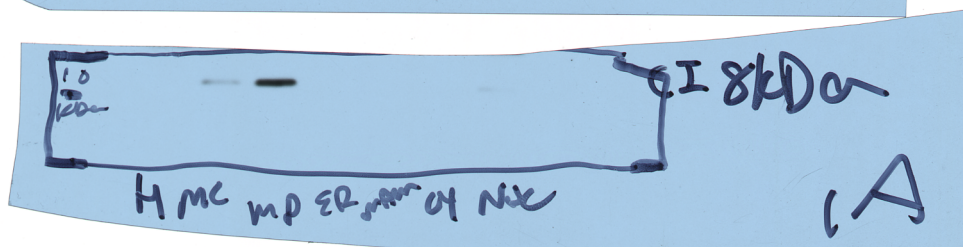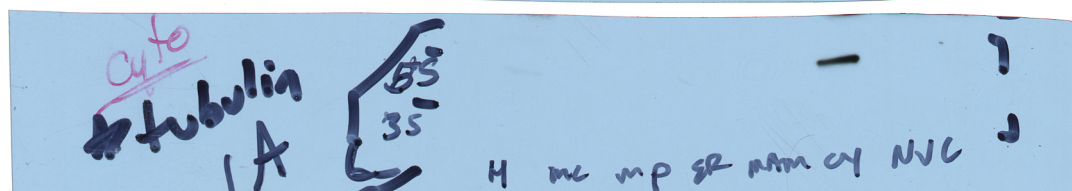

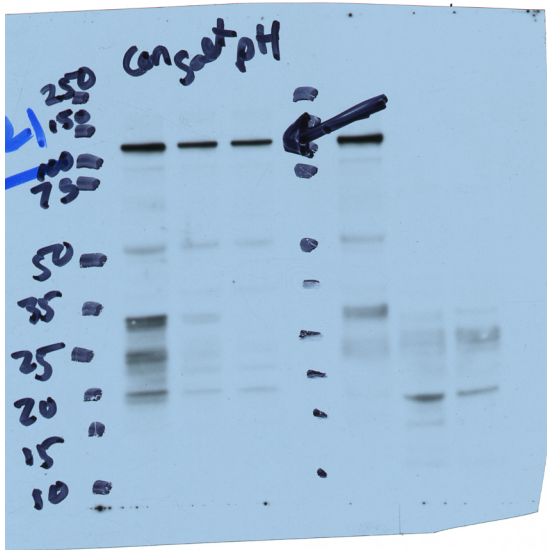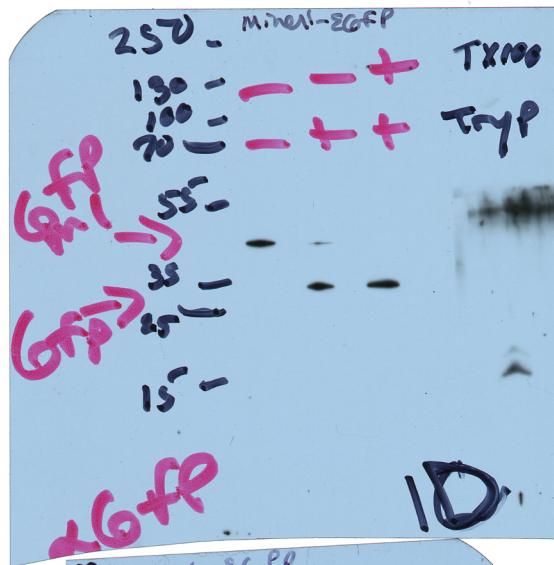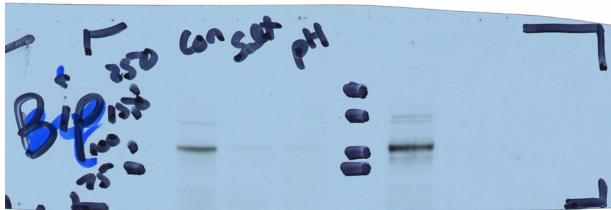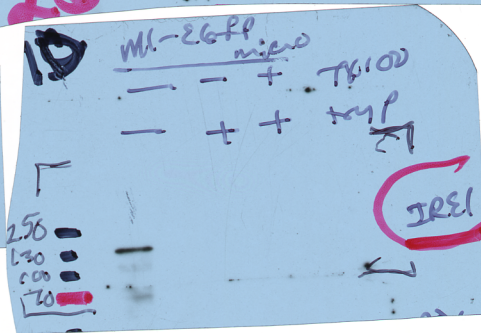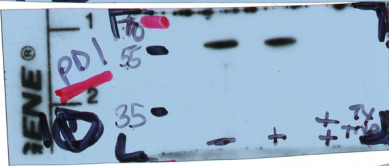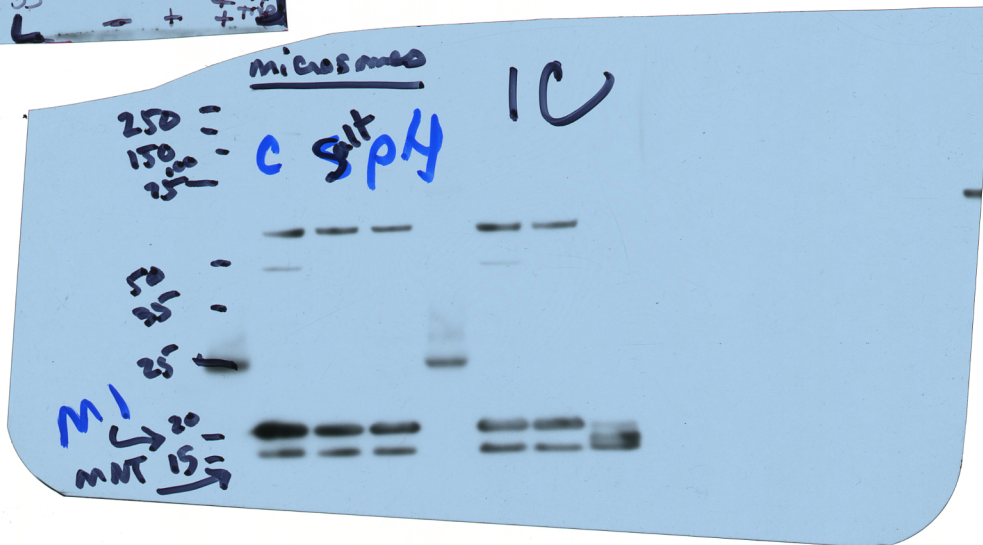

Supplement: Supplementary file 2 [file emmm0005-0904-SD2.pdf]

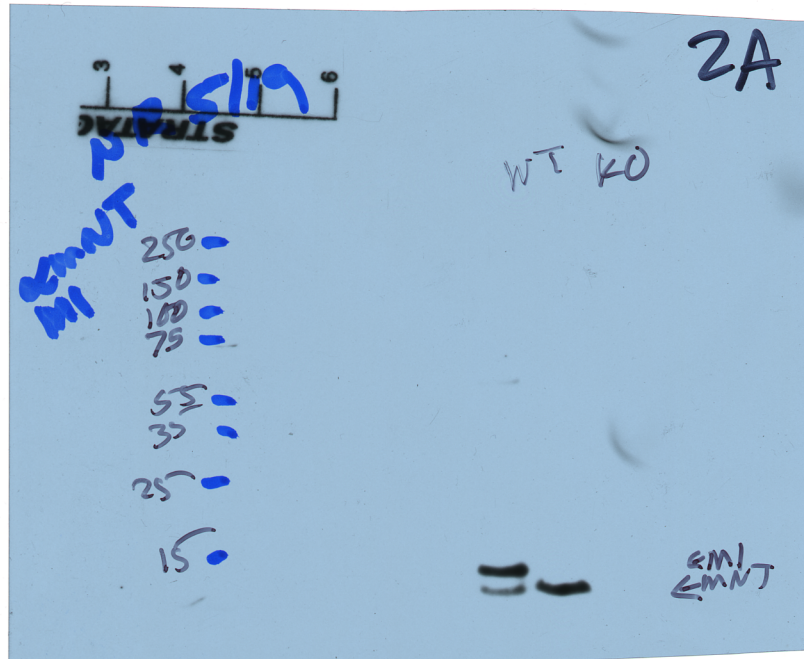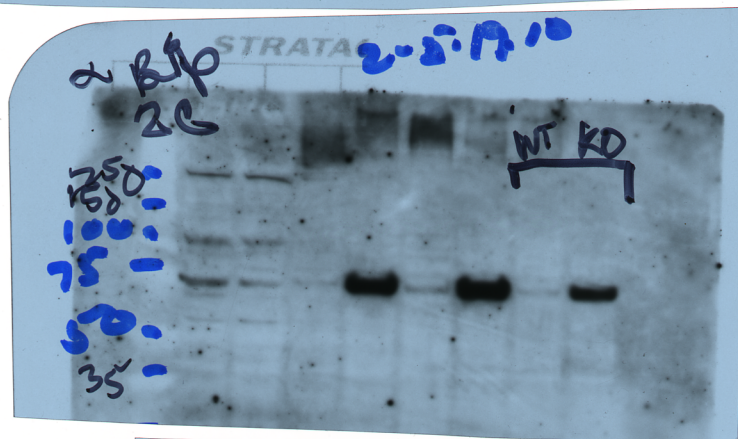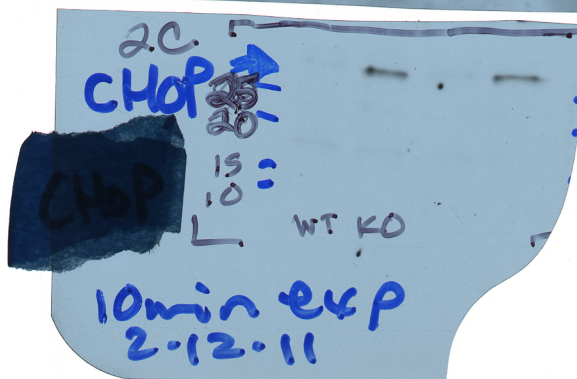

Supplement: Supplementary file 3 [file emmm0005-0904-SD3.pdf]

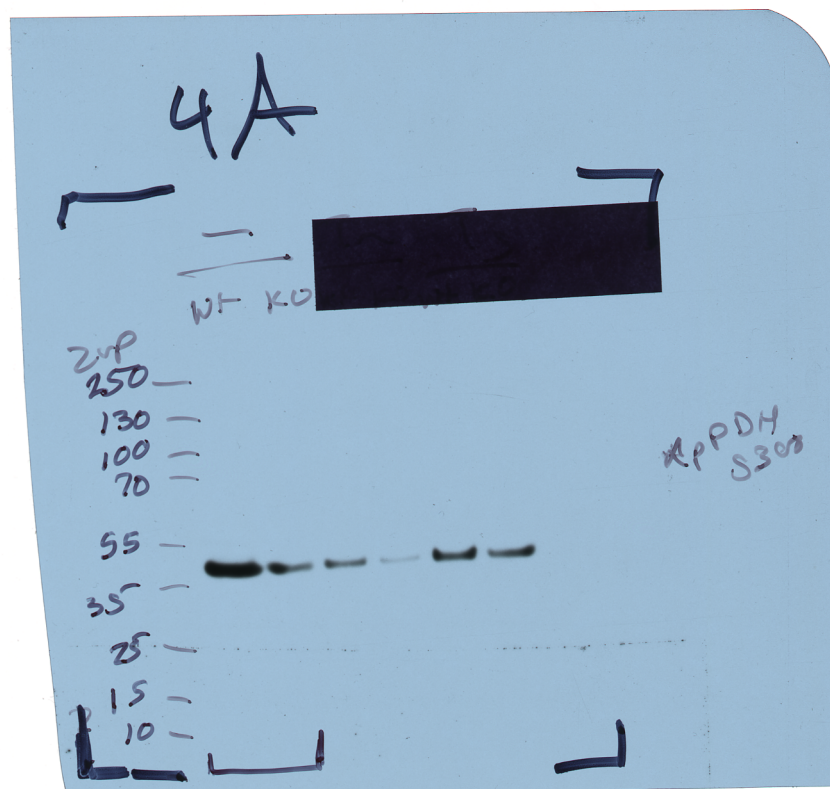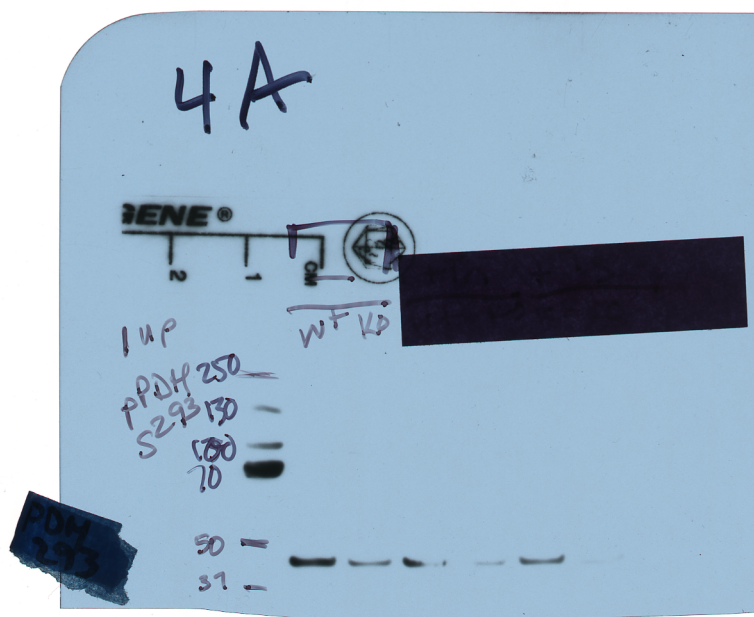

Supplement: Supplementary file 4 [file emmm0005-0904-SD4.pdf]

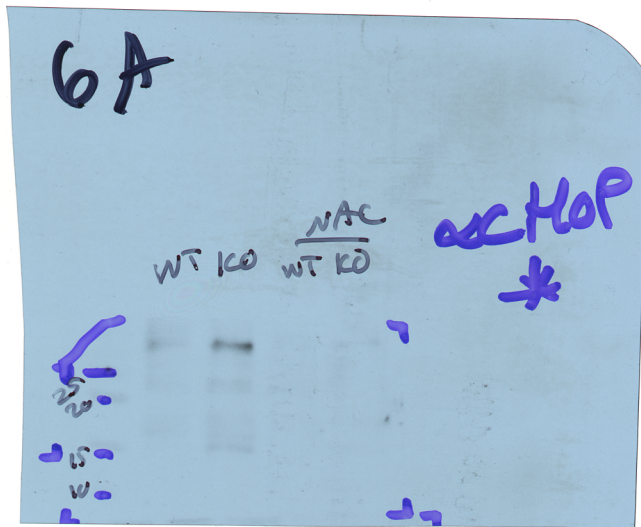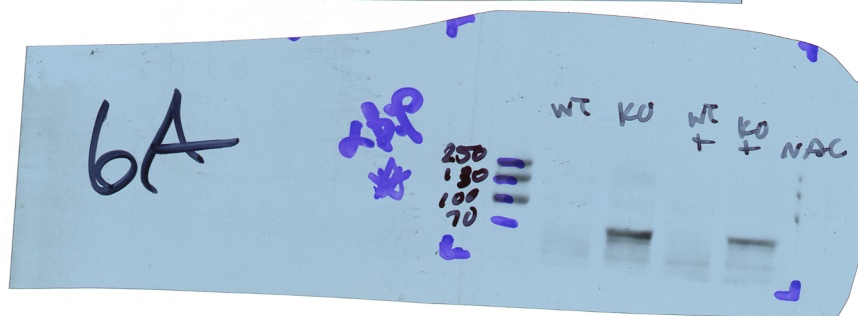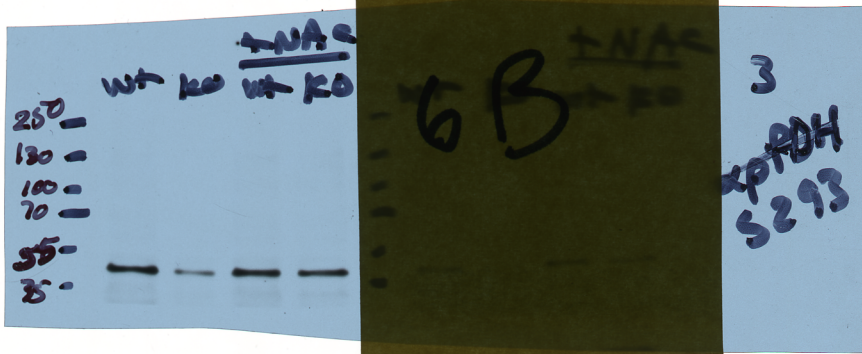

Supplement: Supplementary file 5 [file emmm0005-0904-SD5.pdf]
