# Supplementary material for: Wolfram Syndrome protein, Miner1, regulates sulphydryl redox status, the unfolded protein response, and Ca2+ homeostasis
Source: EMBO Mol Med. 2013 May 24;5(6):904–18. doi: 10.1002/emmm.201201429 (PMC3779451; doi:10.1002/emmm.201201429)
Supplement: Supplementary file 6 [file emmm0005-0904-SD6.pdf]

Supporting Information

Wolfram Syndrome protein, Miner1, regulates sulfhydryl redox status, the unfolded protein response and Ca^{2+} homeostasis

Sandra E. Wiley, Alexander Y. Andreyev, Ajit S. Divakaruni, Robert Karisch, Guy Perkins, Estelle A. Wall, Peter van der Geer, Yi-Fan Chen, Ting-Fen Tsai, Melvin I. Simon, Ben Neel, Jack E. Dixon, Anne N. Murphy

Supporting Information: Results

1. **Supporting Information Fig 1.** Triple stain immunofluorescence of Miner1 localization.

2. **Supporting Information Fig 2.** Triple stain immunofluorescence of GFP localization.
3. **Supporting Information Fig 3.** Assessment of Ca^{2+} content of intracellular Ca^{2+} stores.
4. **Supporting Information Fig 4.** Energetic and mitochondrial characterization of Miner1 KO MEFs.
5. **Supporting Information Fig 5.** MitoNEET knockdown in Miner1 KO MEFs.
6. **Supporting Information Fig 6.** Analysis of mitoNEET overexpression in Miner1 WT MEFs.
7. **Supporting Information Fig 7.** Schematic indicating model of dysfunction observed in Miner1 deficient MEFs and relationship to underlying pathology of Wolfram Syndrome (DIDMOAD).
8. **Supporting Information Table 1.** Oxidation of Catalytic Cys in PTPs

Supporting Information: Methods

Supporting Information: References

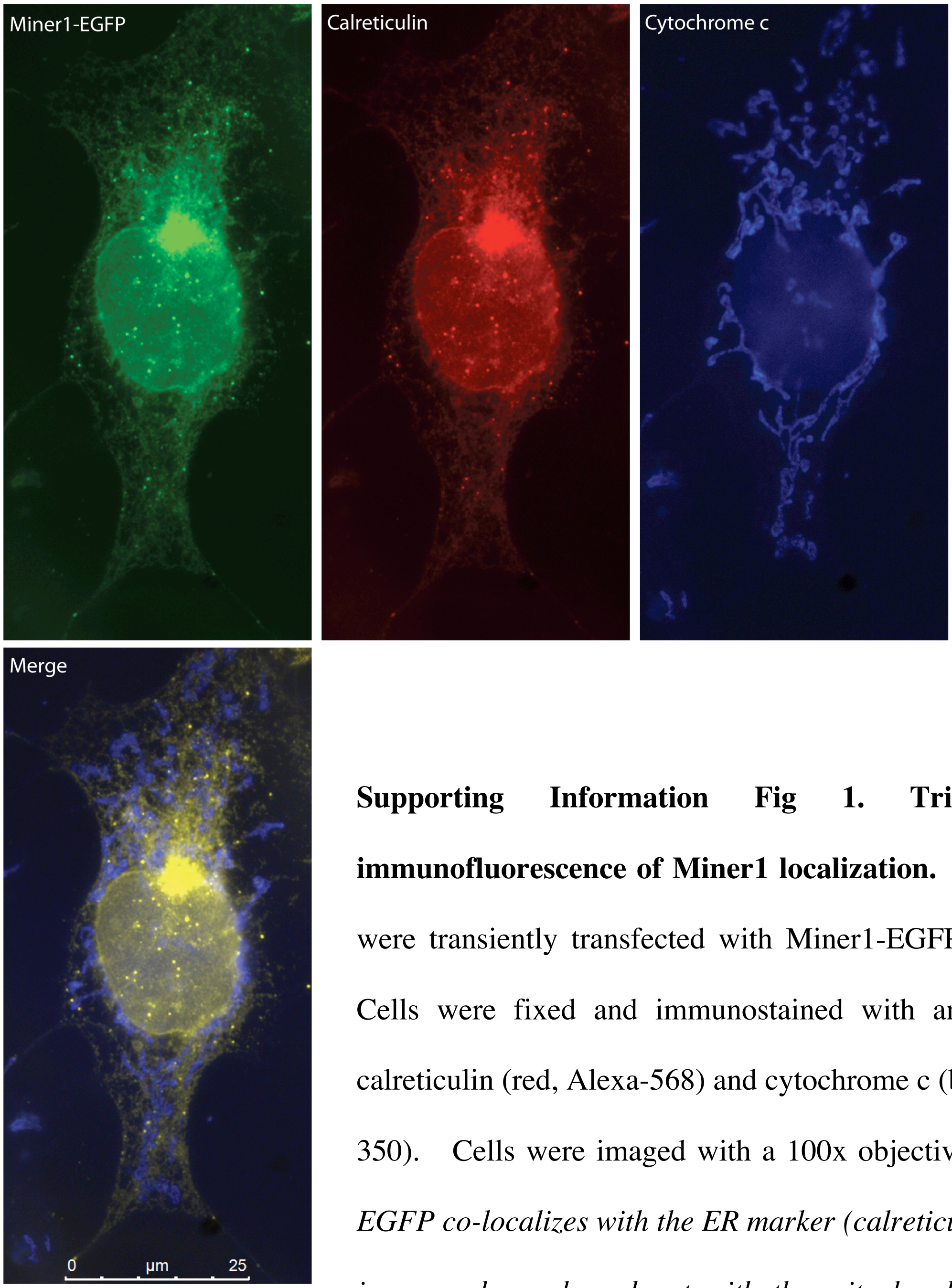

Supporting Information Fig 1. Triple stain immunofluorescence of Miner1 localization. U2OS cells were transiently transfected with Miner1-EGFP for 48 hr. Cells were fixed and immunostained with antibodies to calreticulin (red, Alexa-568) and cytochrome c (blue, Alexa-350). Cells were imaged with a 100x objective. *Miner1-EGFP co-localizes with the ER marker (calreticulin), yellow in merged panel, and not with the mitochondrial marker (cytochrome c).* EGFP controls are shown in Supporting Fig 2.

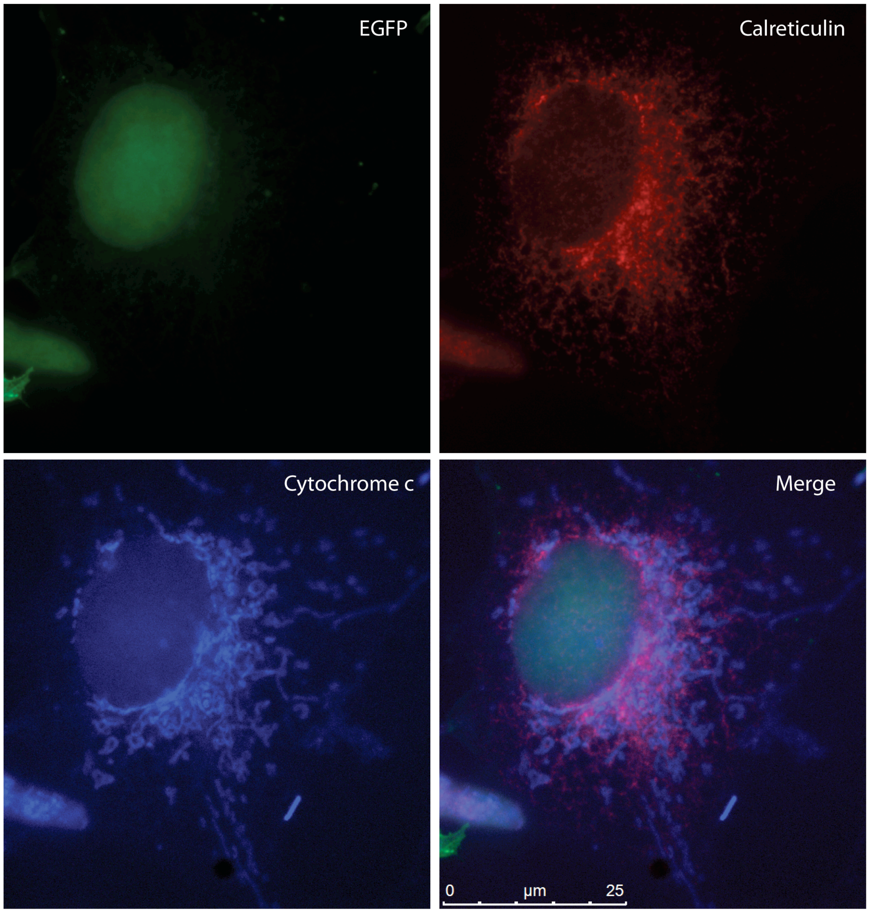

Supporting Information Fig 2. Triple stain immunofluorescence of EGFP localization. U2OS cells were transiently transfected with EGFP for 48 hr, as a control for the images shown in Supporting Fig 1. Cells were fixed and immunostained with antibodies to calreticulin (red, Alexa-568) and cytochrome c (blue, Alexa-350). Cells were imaged with a 100x objective.

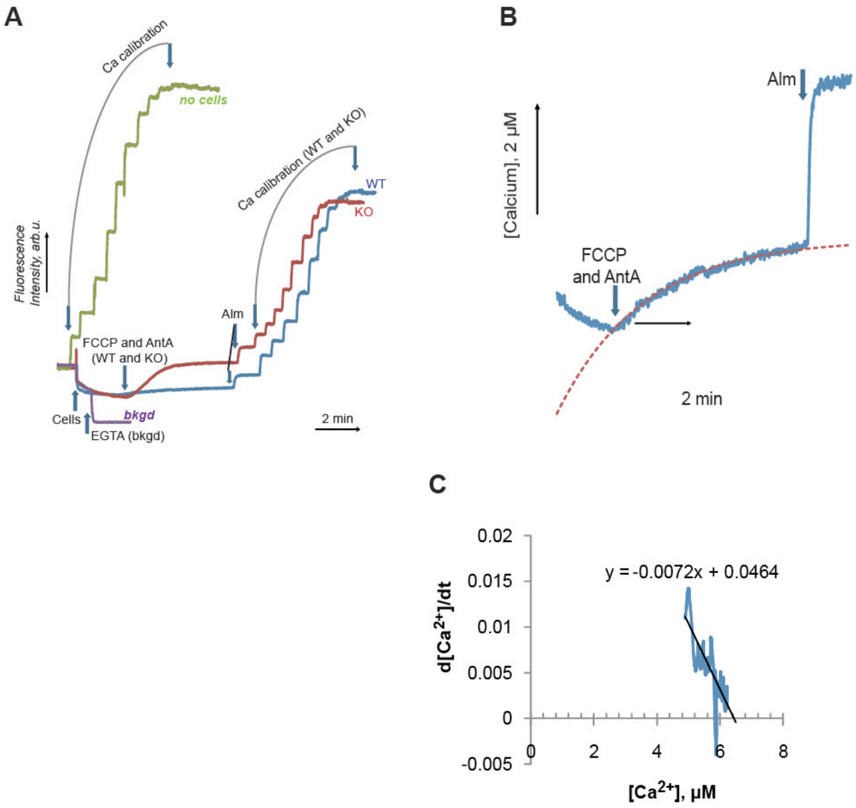

Supporting Information Fig 3. Assessment of Ca^{2+} content of intracellular Ca^{2+} stores.

A. A representative experiment with WT and KO cells (5x10^{6} digitonin-permeabilized MEFs/ml) with: FCCP (1 µM), AntA (0.5 µM), Alm (80 µg/ml), Ca^{2+} calibration. **B.** Curve fitting for the assessment of mitochondrial Ca^{2+}. Dashed line shows regression fit of Ca^{2+} release curve for WT MEFs (panel A) according to first order kinetics. **C.** Linearization of the Ca^{2+} release curve according to first-order mass action law. Black line shows result of linear regression of $d[Ca^{2+}]/dt$ *vs* $[Ca^{2+}]$ plot (blue curve); x-axis intersect represents total (FCCP+AntA-releasable) Ca^{2+}. See Supporting Methods for more detail.

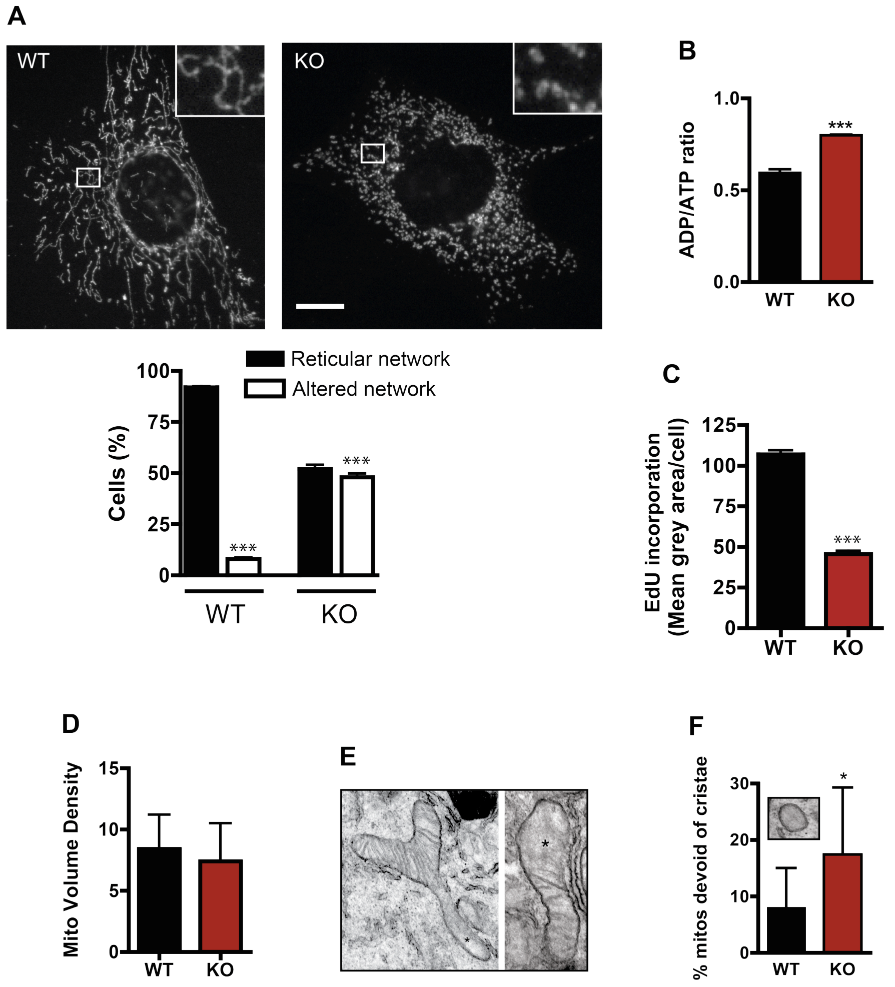

Supporting Information Fig 4. Energetic and mitochondrial characterization of Miner1 KO MEFs.

A. Anti-cytochrome c immunofluorescent analysis of WT and KO MEFs, showing representative images of normal reticular mitochondrial network as well as more punctate altered mitochondrial network. (ANOVA, n=4 experiments, 70-300 cells WT and KO per experiment. (asterisk denotes p<0.0001) Scale bar is 10μm. **B.** ADP/ATP ratios from WT and KO MEFs (t-test, n=4, p<0.0001). **C.** EdU incorporation into DNA to monitor DNA synthesis, fluorescence expressed as mean grey value intensity (t-test, n=3, p< 0.0001). **D.** Mitochondrial volume density (volume of mitochondria/volume of cytoplasm) in WT and KO MEFs was determined from EM micrographs, no significant difference. **E.** Example EM micrographs from Miner1 KO MEFs demonstrating mitochondrial extensions and areas devoid of cristae (marked with asterisk). **F.** EM micrograph of KO mitochondria devoid of cristae. Quantitation of % mitochondria devoid of cristae in WT and KO cells, based on EM micrograph analysis (t-test, p=0.037).

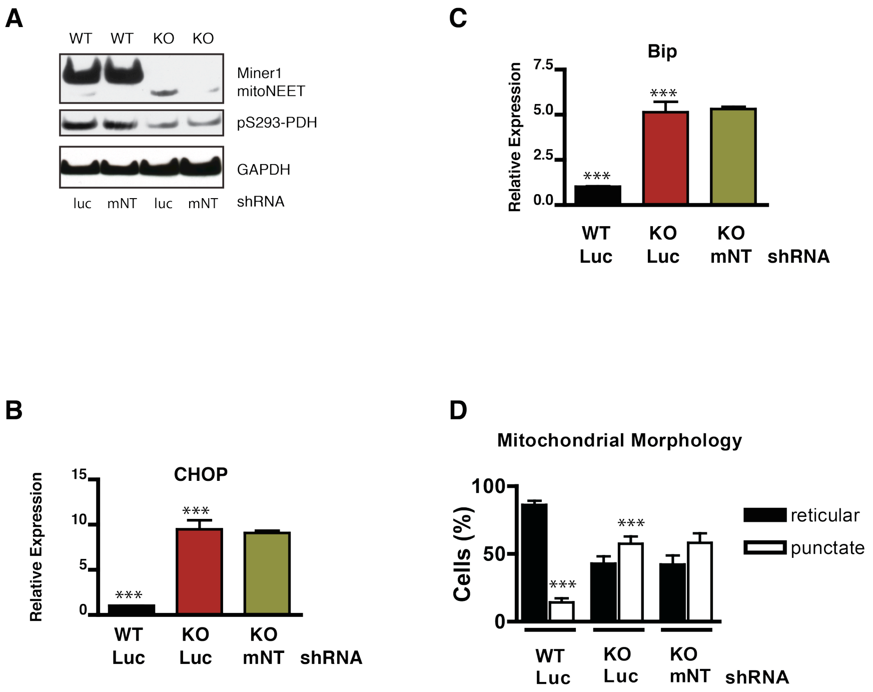

Supporting Information Fig 5. Analysis of mitoNEET knockdown in Miner1 KO MEFs.

A. Western blot analysis of WT and Miner1 KO MEF cell lines expressing an shRNA to either mitoNEET (mNT) or luciferase (Luc) control. Cell extracts (30 μg protein) were blotted for each. Reduction of mitoNEET in Miner1 KO cells does not change pS293-PDH levels. **B. & C.** qRT-PCR analysis of cells described in (A), (t-test, n=3 for each.) Knockdown of mitoNEET in Miner1 KO cells does not alter mRNA levels of UPR marker genes relative to KO Luc cells. Asterisks signify p<0.001 for WT Luc versus KO Luc. **D.** Mitochondrial morphology determined from anti-cytochrome c immunofluorescent analysis. (ANOVA, n=3 experiments, 80-200 cells WT and KO per experiment. Reduction of mitoNEET in Miner1 KO cells does not change mitochondrial morphology relative to KO Luc cells. Asterisks signify p<0.001 for WT Luc versus KO Luc. For B,C,D, there is not a significant difference between KO Luc and KO mNT shRNA samples. *Thus, elevated mitoNEET protein levels in Miner1 KO cells do not appear to be responsible for phenotype observed in the KO cells.*

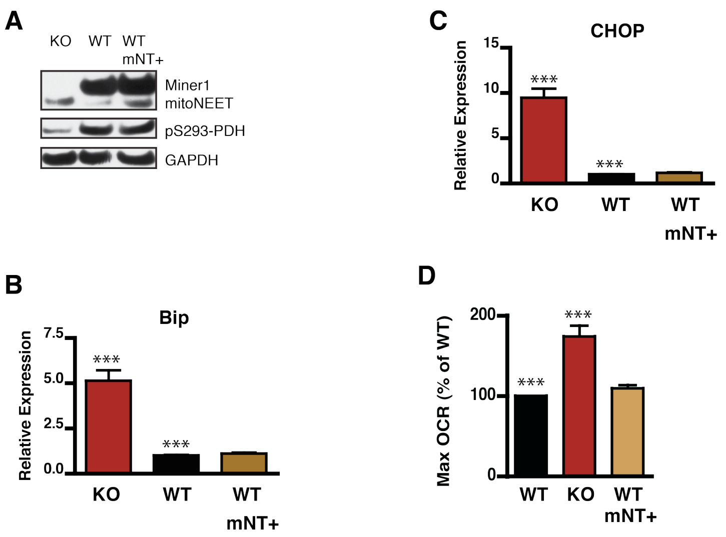

Supporting Information Fig 6. Analysis of mitoNEET overexpression in Miner1 WT MEFs.

A. Western blot analysis of Miner1 KO and WT MEF cell lines, including WT MEFs exogenously overexpressing mitoNEET (mNT+). Cell extracts (30 μg protein) were blotted for each. Increasing mitoNEET in Miner1 WT cells does not change pS293-PDH levels. **B. & C.** qRT-PCR analysis of cells described in (A), (ANOVA, n=3, asterisks signify p<0.001 for KO versus WT). Increasing mitoNEET in Miner1 WT cells does not alter mRNA levels of UPR marker genes, relative to WT cells. **D.** Oxygen consumption rates of cells described in (A) were measured in a Seahorse XF24. Maximal FCCP-stimulated rates relative to WT are shown, (ANOVA, n=6, asterisks signify p<0.001 for KO versus WT. For B, C, D, there is not a significant difference between WT and WT mNT+ samples. *Thus, elevated mitoNEET protein levels in Miner1 KO cells do not appear to be responsible for phenotype observed in the KO cells.*

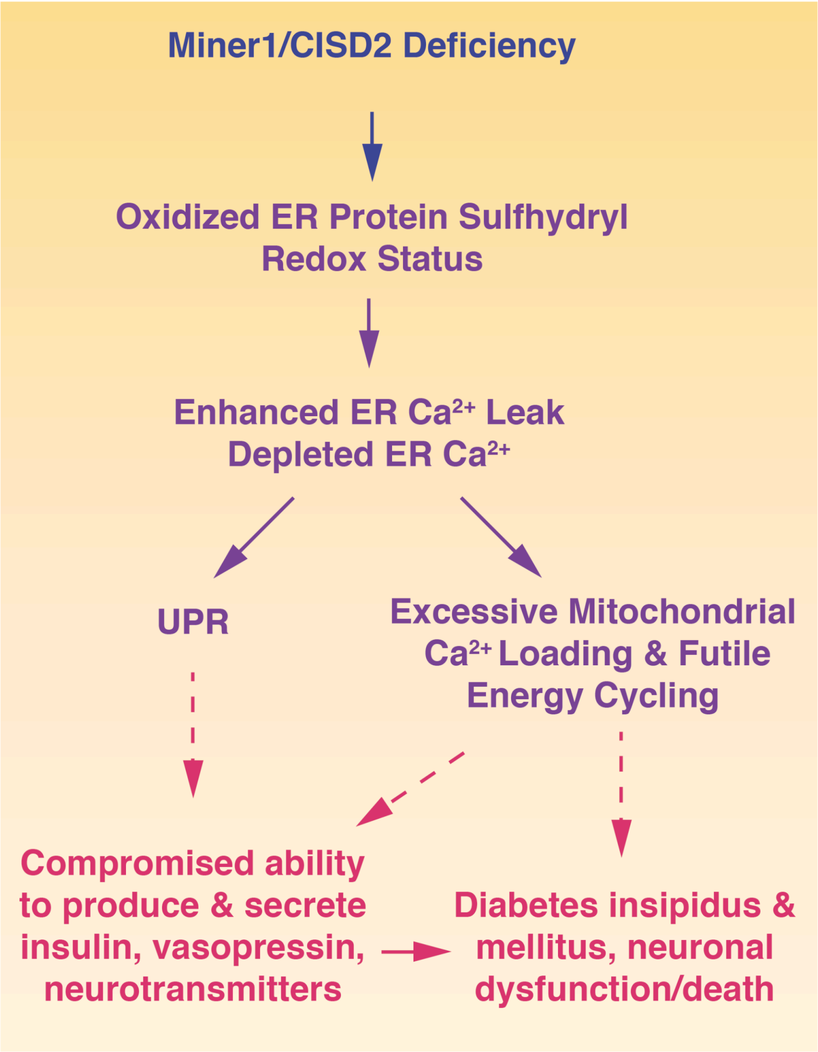

Supporting Information Fig 7. Schematic indicating model of dysfunction observed in Miner1 deficient MEFs and relationship to underlying pathology of Wolfram Syndrome (DIDMOAD).

Solid lines (purple) reflect data presented, whereas dashed lines (red) represent inferred connections to disease pathology.

Relative % Oxidized PTPs

PTP	WT<br>% ox	KO<br>% ox	Total Intensity	Significant Change
Ptpn1	2.6	3.5	2.25×10^7	*
Ptpn2	0.0	0.0	2.51×10^7	
Ptpn4	0.0	5.5	1.56×10^6	*
Ptpn9	0.0	0.0	1.20×10^6	
Ptpn11	2.2	2.4	2.25×10^7	
Ptpn12	4.5	13.0	4.74×10^6	*
Ptpn14	0.0	10.7	1.59×10^6	*
Ptpn21	9.1	55.5	3.52×10^6	*
Ptpn23	100.0	100.0	5.95×10^6	
PtpraD1	12.2	8.1	2.78×10^6	*
PtpraD2	98.9	83.7	1.31×10^7	
PtpreD1	15.2	25.8	4.04×10^6	
PtpreD2	42.1	44.6	4.07×10^6	
PtprfD2	21.3	12.9	1.08×10^6	*
PtprkD1	82.5	100.0	5.85×10^6	*
PtprsD1	0.0	0.0	9.26×10^6	

Supporting Information Table 1. Oxidation of Catalytic Cysteines in PTPs.

Relative oxidation of various PTPs (expressed as a % of total) in WT and Miner1 KO MEFs as detected by Mass Spectrometry following immunoprecipitation with an OX-PTP antibody. Only PTPs with a total intensity $\geq 1.0 \times 10^6$ are shown.

Supporting Information: Methods

Cell culture and reagents.

Miner1 (Cisd2) WT and KO MEFs were obtained from the Tsai lab in Taiwan (Chen et al, 2009). Cells at passage 2 were immortalized using the pBabepuro SV40 tT retrovirus expressing T antigen, which had been packaged in HEK293T cells with gagpol and VSVG packaging plasmids. MEFs were maintained in a humidified incubator at 37°C with 5% CO_2. Cells were initially cultured in DMEM with high glucose (Gibco) supplemented with 20% FBS, 5 mM Gln, and 1 μg/ml puromycin. After 3 passages, the cells were cultured in DMEM with 10% FBS, 5 mM Gln, 100 units/ml penicillin, and 100 mg/ml streptomycin (Gibco). HEK293T cells were also cultured in this media. HEK293T cells were transfected with plasmids encoding either Miner1-GFP (Wiley et al, 2007a) or GFP-Miner1 (Chen et al, 2009) using Fugene (Roche). Cells were harvested for microsomal isolation or fixed for immunofluorescent staining 24-48 h post transfection. WT and KO MEFs expressing shRNA to either luciferase (Luc) or mitoNEET (mNT) were created by transducing cells with pSLIK shRNA-containing lentiviruses as described (Shin et al, 2006) and stable populations selected as neomycin resistant pools. mitoNEET over-expressing WT cells were created by transfection with pcDNA3.1+ plasmid encoding the human mitoNEET CDS using Xtreme Gene9 (Roche); stable populations were selected as neomycin resistant pools. Miner1 "rescue" cells were created by lentivirus-mediated transduction of Miner1 KO cells. Stable populations were selected as neomycin resistant pools.

Tissue/cell fractionation and manipulation.

For subcellular fractionation of rat liver into crude mitos, pure mitos, ER, MAMs, and cytosolic/soluble enriched samples, the protocol of Wieckowski was followed (Wieckowski et al, 2009). Equal amounts of protein from each fraction were separated by SDS-PAGE and analyzed by Western blotting. Mitochondria and ER enriched fractions were prepared from fresh rat livers as previously described (Wiley et al, 2007b). Treatment of rat liver microsomes with high salt (200 mM KCl), high pH (100 mM Na_2CO_3, pH 11), and trypsin was done as previously described (Wiley et al, 2007b). For isolating mitochondria from Miner1 WT and KO MEFs, cells were lysed using nitrogen cavitation (10 min at 9,000 psi), and the mitochondria purified by differential centrifugation as described (Wiley et al, 2007b). Microsomes from HEK293T cells transiently transfected with Miner1-EGFP were isolated as follows: cells were lysed in STKM buffer (0.2 M sucrose, 0.05 M tris/HCl, pH 7.5, 0.05 M KCl, 0.005 M $MgCl_2$), subjected to nitrogen cavitation, centrifuged at 3,500 x g to pellet nuclei, then the supernatant centrifuged at 9,000 x g to pellet the mitochondria and ER-associated MAMs, after which the supernatant was centrifuged at 100,000 x g to pellet the microsomes. The microsomes were then treated with trypsin as previously described (Wiley et al, 2007b).

Western Blots and immunofluorescence analysis.

In general, cells were lysed in PLC lysis buffer (Copp et al, 2005), insoluble material cleared with a 15,000 x g centrifugation (15 min, 4°C), and protein concentrations of the supernatants determined with the Bradford Protein Assay reagent (Bio-Rad). Equivalent

amounts of proteins were separated on either NUPAGE gels (Invitrogen) or mini-PROTEAN gels (BioRad), transferred to PVDF and subjected to Western blotting with primary antibodies, HRP-conjugated secondary antibodies (Amersham) and ECL detection. For CHOP detection, cells were lysed in RIPA buffer (50 mM Tris, 150 mM NaCl, 1% NP40, 0.5% deoxycholate, 0.1% SDS, 10% glycerol, 0.4 mM EDTA, 100 mM NaF, 500 μM Na vanadate, 10 mM NaPPi). All lysis buffers included Complete Protease inhibitors (Roche). The antibodies used for Western blotting were: SMAC, p-PDH, PDH (EMD Biosciences) (Rardin et al, 2009); Complex I-8 kDa subunit, NDUFB6, CII-30 kDa, ATP Synthase alpha (MitoSciences); Bip, IRE1, CHOP, calnexin (Cell Signaling); GFP (Clontech); GAPDH (Ambion); SERCA2 (Santa Cruz Biotechnology); anti-glutathione (Virogen); tubulin (Sigma-Aldrich). The anti-Miner1/mitoNEET antibody was produced by our laboratory. It was made in rabbits against a recombinant Miner1 CDGSH domain protein. For this, the cDNA encoding amino acids 57-135 of human Miner1 was cloned in frame with a C-terminal 6x His tag into the pET21 vector (EMD Biosciences). Expression and purification was as previously reported for the mitoNEET antibody production (Wiley et al, 2007b).

For immunofluorescence analysis, cells were plated on glass coverslips, fixed with 3.7% paraformaldehyde (PF) and immunostained as described (Wiley et al, 2007b). For cells labeled with MitoTracker Red (Invitrogen), following manufacturer's protocol, the cells were incubated with ice cold 100% methanol for 3 min following the PF fixation. The antibodies used for immunostaining were: calreticulin (EMD Biosciences), cytochrome c (BD Pharmingen), V-5 epitope (Invitrogen). DAPI (Invitrogen) was used

to stain DNA. Cells were imaged with a Leica DMR microscope and a Hamamatsu camera. Images were processed with OpenLab Software.

qRT-PCR gene expression analysis.

RNA was purified using the Qiagen RNA miniprep kit and on-column DNAse treatment. cDNA reactions were performed with the iScript cDNA Synthesis kit (BioRad) using 1 μg of input RNA. qRT-PCR reactions (40 μl) were run in triplicate on an ABI 7500 using Syber Green Master Mix (ABI) and analyzed using the ddCT method. Data are expressed as relative expression (mean fold induction $\pm$ SE). Primers used for qRT-PCR were: Bip (for: 5'-TCATCGGACGCACTTGGAA-3', rev: 5'-ATTGTGGAAGTACGTGAGCTC-3'), CHOP (for: 5'-CCACCACACCTGAAAGCAGAA-3', rev: 5'-GTTGTCGCCAGTCTCTTCG-3'), tXBP1 (for: TGGCCGGGTCTGCTGAGTCCG, rev: GTCCATGGGAAGATGTTCTGG), GAPDH (for: CACCATCTTCCAGGAGCGAG, rev: CCTTCTCCATGGTGGTGAAGAC). Students T-tests and ANOVAs were performed using Prism Graph Pad Software.

Ca^{2+} Assays.

ER Ca^{2+} stores were interrogated *in situ* following 2 μM thapsigargin (Tg)-induced Ca^{2+} release using the cell-permeable fluorescent ratiometric dye Fura2-AM (Invitrogen) and a FLEX-Station (Molecular Devices) in a 96 well plate format (30,000 cells/well plated the day before) with 3-6 replicates per experiment. For the Miner1 rescue experiments in Fig.7, 60,000 cells/well were plated. Alternatively, ER Ca^{2+}

release *via* the IP3 receptor was triggered by treatment with 100 μM histamine. Minimum and maximum signal intensities were determined for each well during the runs. The procedure and analysis was as per manufacturer's instructions and as described by the AfCS Web site (http://www.signaling-gateway.org/data/ProtocolLinks.html. Procedure Protocol PP00000211). For the Ca^{2+} traces, a representative experiment is shown. Data are expressed as mean of replicate wells $\pm$ SE.

To assess the Ca^{2+} content of mitochondria and other intracellular Ca^{2+} stores, Ca^{2+} was monitored in the incubation medium in the presence of digitonin-permeabilized cells using the membrane-impermeable fluorescent dye Calcium Green 5N (0.5 μM) (Murphy et al, 1996). An LS-50B spectrofluorometer (Perkin-Elmer) was used to monitor dye fluorescence (λ_{EX}=506, λ_{EM}=531). For an experimental run, MEFs were digitonin-permeabilized as follows: 10^7 cells were pelleted from ~ 1 ml aliquot (200 g, 2 min), washed with 1 ml of medium containing 250 mM sucrose, 2 mM potassium phosphate and 20 mM HEPES-KOH, pH 7.0, pelleted again and permeabilized by resuspending in 0.5 ml of incubation medium (125 mM KCl, 2 mM potassium phosphate, 20 mM HEPES-KOH, pH 7.0, 1 mM $MgCl_2$, 5 mM glutamate and 5 mM malate) supplemented with 0.5 μl of 10% digitonin (0.01% final concentration). The final concentration of cells was $5x10^6$ digitonin-permeabilized MEFs/ml.

Mitochondrial Ca^{2+} uptake and Ca^{2+} release were detected as a fluorescence decrease upon addition of permeabilized cells and as a fluorescence increase upon addition of Ca^{2+}-releasing agents, respectively (Supporting Information Fig 3A). Mitochondrial Ca^{2+} is selectively released through a reversal of the uptake mechanism (Ca^{2+} uniporter) upon addition of agents that depolarize mitochondria. We used a

combination of uncoupler (1 μM FCCP) and respiratory inhibitor (0.5 μM antimycin A, AntA) (Kushnareva et al, 2005). It should be noted that this process is fairly slow (Supporting Information Fig 3B), which necessitates additional calculations to obtain precise values (see below). To completely empty all intracellular stores, including ER, we used alamethicin (Alm, 80 μg/ml), which forms large non-selective pores in phospholipid membranes.

The amount of released Ca^{2+} was calculated based upon calibration of the fluorescent signal by additions of known concentrations of $CaCl_2$ to the medium (sequential additions of $CaCl_2$ starting from 5 μM and doubled with each subsequent addition up to 2560 μM total). The fluorescent signal is influenced by the presence and type of cells. As evident from Supporting Information Fig 3A, completely saturated signal (at 1280 and 2560 μM of added Ca^{2+}) is reduced by the presence of cells ("no cells" *vs* "WT" *vs* "KO"). Therefore, the calculations had to be based upon internal calibrations included at the end of each experimental run. Background fluorescence was established by chelating contaminating Ca^{2+} with EGTA (trace "bkgd") and subtracted from all measured fluorescence values.

Due to the presence of released Ca^{2+}, this calibration deviates from theoretical (hyperbolic) binding curve; however, incremental increase of added Ca^{2+} levels by the amount of released Ca^{2+} restores the theoretical shape of the calibration curve. This way, the amount of released Ca^{2+} ($[Ca^{2+}]_{released}$) was determined in the regression analysis of the calibration curve.

$$I = I_{max}*([Ca^{2+}]_{added} + [Ca^{2+}]_{released}) / K_d^{app} + ([Ca^{2+}]_{added} + [Ca^{2+}]_{released}),$$

where I is dependent variable (fluorescence intensity in the presence of a known concentration of added Ca^{2+}), $[Ca^{2+}]_{added}$ is independent variable (concentration of added Ca^{2+}), and apparent binding constant K_d^{app}, maximal fluorescence I_{max}, and $[Ca^{2+}]_{released}$ are regression parameters to be determined.

Regression parameters K_d^{app} and I_{max} were further used for conversion of fluorescence signal to Ca^{2+} concentrations (shown on panel B). It should be noted, that the apparent K_d (K_d^{app}) values as determined in this assay (37-44 μM) are substantially different from the true binding constant reported by the manufacturer (Molecular Probes, 14 μM); therefore, the latter cannot be used in place of the measured values.

As evident from a typical curve (Supporting Information Fig 3B), mitochondrial Ca^{2+} release may not reach completion in the time frame of the experiment. To obtain precise values of mitochondrial Ca^{2+}, an analysis of complete kinetic curves was performed based on the assumption of first-order kinetics (Supporting Information Fig 3C). For this analysis, Ca^{2+} release curves were first smoothed using moving average (100 points = 10 s). Then, Ca^{2+} release rate ($d[Ca^{2+}]/dt$) was determined at each point by performing linear regression of $[Ca^{2+}]$ *vs* time data using 200 subsequent points (20 s). According to mass action law

$$d[Ca^{2+}]/dt = -k*[Ca^{2+}]$$

$d[Ca^{2+}]/dt$ data plotted *vs* Ca^{2+} concentration form a straight line. The x-axis intersect of this plot is the Ca^{2+} concentration at which Ca^{2+} release rate equals 0, *i.e.*, the release is

complete. Therefore, the x-axis intersect represents the precise value of Ca^{2+} quantity releasable by the mitochondrial inhibitors FCCP + AntA (mitochondrial Ca^{2+}).

Both mitochondrial and total intracellular Ca^{2+} (releasable by alamethicin) determined in these experiments had to be corrected for the initial uptake of contaminating Ca^{2+} from the medium (Supporting Information Fig 3A). Contaminating Ca^{2+} for each experimental run was recorded prior to the addition of cells and, therefore, was calculated as described above but using a separate "no cells" calibration curve (Supporting Information Fig 3A). Subsequently, the contaminating Ca^{2+} levels were subtracted from the corresponding mitochondrial and total Ca^{2+} levels to obtain net mitochondrial and total intracellular store content (Fig. 3D). It should be noted that the mitochondrial Ca^{2+} content in WT cells is very low and the amount of released Ca^{2+} is approximately the same magnitude as the contaminating Ca^{2+} in these cells. This can result in a slightly negative value for mitochondrial Ca^{2+} in WT cells after the background is subtracted.

Mitochondrial respiration, ATP and cell proliferation measurements.

The Seahorse XF24 Extracellular Flux Analyzer was used to determine OCR as a measure of mitochondrial respiratory function. When confluent, the Miner1 KO MEFs tended to lift off of the Seahorse plates due to the mixing during the run. To circumvent this problem, cells (100,000/well) were adhered to the Seahorse plates using Cell-Tak (BD Biosciences) immediately before analysis. Whole cells were analyzed in unbuffered DMEM supplemented with 10 mM glucose, 10 mM pyruvate, and 2 mM glutamine (Zhang et al, 2011). Oligomycin (1 μM) was used to inhibit the ATP Synthase; FCCP

(carbonylcyanide-p-trifluoromethoxyphenylhydrazone) was titrated (0.6-1.2 μM) to measure maximal uncoupler-stimulated OCR; Rotenone (100 nM) and antimycin A (2 μM) were used to inhibit all mitochondrial respiration and monitor non-mitochondrial oxygen consumption. OCR in mitochondria isolated from WT and Miner1 KO MEFs that were spun onto assay plates was performed in the Seahorse XF24 (10 μg/well) with specific oxidizable substrates (either 10mM glutamate/10mM malate or 10 mM succinate/2 μM rotenone, which supply reducing equivalents to Complex I and Complex II of the ETC, respectively) following the protocol previously described (Rogers et al, 2011). For antioxidant treatment, WT and KO cells were treated with 5 mM NAC in cell culture dishes for 48 hrs, after which they were harvested, adhered to Seahorse plates using Cell-Tak, and immediately analyzed. OCR replicates varied from 3 to 6 per experiment. Unless stated otherwise, all chemicals were from Sigma-Aldrich. ADP/ATP ratios were determined using the fluorescent ApoSensor ADP/ATP Ratio kit (BioVision). ATP concentrations relative to cell number were measured using the Cell-Titer Glo kit (Promega) following manufacturer's instructions, followed by normalization to propidium iodide fluorescence per well. Values expressed as luminescence (cps)/fluorescence (RFU). Cell proliferation was assessed by EdU (5-ethynyl-2'-deoxyuridine) incorporation into DNA using the Click-iT EdU Imaging kit (Invitrogen) to monitor DNA synthesis. Cells were labeled for 2 hrs with EdU (Invitrogen), followed by Alexa Fluor-488 azide, and visualized utilizing fluorescent microscopy. Images were captured at identical camera settings, followed by analysis with Image J software. Data from 3 experiments are represented with 30 – 70 cells measured per experiment ($p < 0.0001$).

Analysis of CX_5R PTP oxidation state.

For oxidized PTP blots, cells were lysed in PLC lysis buffer with 3 mM DTT, cleared of insoluble material, and lysates were analyzed by SDS-PAGE with an antibody recognizing the sulfonic acid oxidation state of the active site cysteine (R&D Systems). Samples were prepared and analyzed as previously reported (Karisch et al, 2011). Briefly, WT and Miner1 KO MEFs were lysed in an NEM (N-ethyl-maleimide) containing buffer to alkylate all of the reduced cysteines. Following this, any partially oxidized cysteines were fully oxidized to the sulfonic acid state using oxidants, followed by immunoprecipitation with the OX-PTP antibody and analysis by mass spectrometry.

Supporting Information: References

Chen Y-F, Kao C-H, Chen Y-T, Wang C-H, Wu C-Y, Tsai C-Y, Liu F-C, Yang C-W, Wei Y-H, Hsu M-T, Tsai S-F, Tsai T-F (2009) Cisd2 deficiency drives premature aging and causes mitochondria-mediated defects in mice. *Genes & Development* **23:** 1183-1194

Copp J, Wiley S, Ward MW, van der Geer P (2005) Hypertonic shock inhibits growth factor receptor signaling, induces caspase-3 activation, and causes reversible fragmentation of the mitochondrial network. *Am J Physiol Cell Physiol* **288:** C403-415

Karisch R, Fernandez M, Taylor P, Virtanen C, St-Germain JR, Jin LL, Harris IS, Mori J, Mak TW, Senis YA, Ostman A, Moran MF, Neel BG (2011) Global proteomic assessment of the classical protein-tyrosine phosphatome and "Redoxome". *Cell* **146:** 826-840

Kushnareva YE, Wiley SE, Ward MW, Andreyev AY, Murphy AN (2005) Excitotoxic injury to mitochondria isolated from cultured neurons. *J Biol Chem* **280:** 28894-28902

Murphy AN, Bredesen DE, Cortopassi G, Wang E, Fiskum G (1996) Bcl-2 potentiates the maximal calcium uptake capacity of neural cell mitochondria.

Proceedings of the National Academy of Sciences of the United States of America **93:** 9893-9898

Rardin MJ, Wiley SE, Naviaux RK, Murphy AN, Dixon JE (2009) Monitoring phosphorylation of the pyruvate dehydrogenase complex. *Analytical Biochemistry* **389:** 157-164

Rogers GW, Brand MD, Petrosyan S, Ashok D, Elorza AA, Ferrick DA, Murphy AN (2011) High throughput microplate respiratory measurements using minimal quantities of isolated mitochondria. *PLoS ONE* **6:** e21746

Shin KJ, Wall EA, Zavzavadjian JR, Santat LA, Liu J, Hwang JI, Rebres R, Roach T, Seaman W, Simon MI, Fraser ID (2006) A single lentiviral vector platform for microRNA-based conditional RNA interference and coordinated transgene expression. *Proceedings of the National Academy of Sciences of the United States of America* **103:** 13759-13764

Wieckowski MR, Giorgi C, Lebiedzinska M, Duszynski J, Pinton P (2009) Isolation of mitochondria-associated membranes and mitochondria from animal tissues and cells. *Nat Protoc* **4:** 1582-1590

Wiley SE, Murphy AN, Ross SA, van der Geer P, Dixon JE (2007a) MitoNEET is an iron-containing outer mitochondrial membrane protein that regulates oxidative capacity. *Proceedings of the National Academy of Sciences of the United States of America* **104:** 5318-5323

Wiley SE, Murphy AN, Ross SA, van der Geer P, Dixon JE (2007b) MitoNEET is an iron-containing outer mitochondrial membrane protein that regulates oxidative capacity. *Proc Natl Acad Sci U S A* **104:** 5318-5323

Zhang J, Guan Z, Murphy AN, Wiley SE, Perkins GA, Worby CA, Engel JL, Heacock P, Nguyen OK, Wang JH, Raetz CR, Dowhan W, Dixon JE (2011) Mitochondrial phosphatase PTPMT1 is essential for cardiolipin biosynthesis. *Cell Metabolism* **13:** 690-700
